# Artificial Neural Network Correlation and Biostatistics Evaluation of Physiological and Molecular Parameters in Healthy Young Individuals Performing Regular Exercise

**DOI:** 10.3389/fphys.2019.01242

**Published:** 2019-10-02

**Authors:** Kitti Garai, Zoltan Adam, Robert Herczeg, Emese Katai, Tamas Nagy, Szilard Pal, Attila Gyenesei, Judit E. Pongracz, Marta Wilhelm, Krisztian Kvell

**Affiliations:** ^1^Department of Pharmaceutical Biotechnology, Faculty of Pharmacy, University of Pécs, Pécs, Hungary; ^2^Wnt Signaling Research Group, Szentagothai Research Center, University of Pécs, Pécs, Hungary; ^3^Bioinformatics Research Group, Szentagothai Research Center, University of Pécs, Pécs, Hungary; ^4^Department of Laboratory Medicine, University of Pécs, Pécs, Hungary; ^5^Department of Pharmaceutical Technology and Biopharmacy, Faculty of Pharmacy, University of Pécs, Pécs, Hungary; ^6^Faculty of Science, Institute of Sport Sciences and Physical Education, University of Pécs, Pécs, Hungary

**Keywords:** aging, physical activity, responsiveness, prediction, prevention

## Abstract

Studies support that regular physical activity (PA) decelerates senescence-related decline of physiological and molecular parameters in the elderly. We have addressed the other end of this spectrum: healthy and young, inactive individuals participated in a 6-month long personal trainer-guided lifestyle program. We have measured physiological and molecular parameters (differentiating high- and low responders) and their correlation with PA (sedentary status). Cluster analysis helped to distinguish individuals with high- or low PA and differentiate high- and low-responders of each parameter. The assessed cardiovascular parameters (heart rate, blood pressure, 6-min walking distance, relative VO_2_max), body composition parameters (body fat and muscle mass percentage) metabolic parameters (glucose, insulin, HDL, LDL), immune parameters (cortisol, CRP, lymphocyte counts, hTREC) all showed improvement. Artificial neural network analysis (ANN) showed correlation efficiencies of physiological and molecular parameters using a concept-free approach. ANN analysis appointed PA as the mastermind of molecular level changes. Besides sedentary status, insulin and hTREC showed significant segregation. Biostatistics evaluation also supported the schism of participants for their sedentary status, insulin concentration and hTREC copy number. In the future ANN and biostatistics, may predict individual responses to regular exercise. Our program reveals that high responder individuals of certain parameters may be low responders of others. Our data show that moderate regular PA is essential to counteract senescence in young and healthy individuals, despite individual differences in responsiveness. Such PA may not seem important in the everyday life of young and healthy adults, but shall become the base for healthy aging.

## Introduction

Statistics show that regular physical activity (PA) helps preventing the development of several chronic diseases including cardiovascular events, malignancies (Beaudry et al., 2018), neurodegenerative conditions (Ahlskog, 2018), diabetes (Yanai et al., 2018) and osteoporosis (Senderovich and Kosmopoulos, 2018). Regular exercise also ameliorates their course and outcome, and physicians often recommend a lifestyle change as adjuvant therapy (Dieli-Conwright et al., 2018). Several studies focus on anthropometric, physiological or molecular parameters showing the positive effects of regular PA that aid the treatment of chronic diseases (Beaudry et al., 2018; Yanai et al., 2018). The beneficial effects of regular exercise were first detected among professional endurance athletes (Fitzgerald, 1988). Later the tendency of positive effects was also measured in healthy, young individuals performing habitual (non-professional) sports (Caglar et al., 2009).

Positive response to exercise is often generalized in literature assuming that the group average represents a typical response for all participants (Bouchard and Rankinen, 2001). However, recently it has also become clear that the human population gives diverse responses even for standardized lifestyle programs (Karavirta et al., 2011). This may be due to differences in genetic background and environmental conditions (nutritional status, sleep quality, psychological status etc.). Adherence to study protocol (compliance) is another significant factor that may show variations during a lifestyle program (Mann et al., 2014). Diversity of molecular parameters occurring during lifestyle studies is still challenging to predict. With these in mind we have examined the effect of a 6 month-long, personal trainer-guided lifestyle program in young and healthy, but previously inactive adults. Daily activity was continuously recorded (using Actigraph device), standard physiological parameters were measured, and 6-min walking tests (6MWT) were performed to validate our program. This was followed by the evaluation of molecular parameters of metabolism and immunity (including corticosteroid hormones, lymphocyte count, and thymus function correlated with lymphocyte count). Computer-based artificial neural network analysis (ANN) revealed correlation patterns between physiological and molecular parameters in a concept-free manner. In addition, we have also performed biostatistics evaluation of the most interconnected and deregulated parameters. Our aim was to differentiate high- and low responders of PA and all the other recorded physiological and molecular factors. Our goal was to identify distinct patterns of responsiveness and segregation that may provide basis for future prediction of molecular level gain.

## Materials and Methods

### Study Participants

The lifestyle program was a university-based trial designed to promote PA among physically inactive university students. The trainings were conducted in the Institute of Sport Sciences and Physical Education. The program was executed from November to May, it was approved by the ethics committee of the University of Pécs (ref. no.: 6439/2016). All participants gave written informed consent before starting the lifestyle program in accordance with the Declaration of Helsinki. All measurements involving human subjects and their blood samples were performed with the consent of the Regional and Local Ethics Committee of Clinical Center, University or Pécs according to their guidelines. We recruited 25 participants altogether via advertisements in social media. The inclusion criteria were: no regular PA or controlled diet program in the previous 6 months, no history of disease (metabolic, cardiovascular, hypertension or major injury). Participants were both males (*n* = 3) and females (*n* = 22), the mean age was 24.95 years (±4.04). At the end of the program 14 participants (male *n* = 2 and female *n* = 12) completed the 6 month-long lifestyle program fulfilling the requirements of attending trainings three times a week.

### Combined Endurance and Strength-Training Program

The lifestyle program was created to upscale both endurance and strength combined into a 60-min exercise session, three times a week, for 6 months. Each session was divided into four stages: 10 min warm-up, 30 min strength training, 20 min aerobic training and 10 min stretching and cool-down. The intensity of PA was adjusted to the actual fitness level of participants: ≤65% HRmax for aerobic exercise and ≤85% HRmax for strength training, where HRmax = 220–age (in years). Every other week training was performed wearing Polar Team system heart rate (HR) monitors. Participants were asked to keep their usual diet and normal daily activity level during the 6-month long lifestyle program.

### Measurements

Measurements were conducted before starting the training program (0 month), after 3- and 6- months. The 3- and 6-month values were normalized to baseline values and converted to Z-scores. Body Fat% and Muscle mass% were measured using Bioimpedance Analyzer (Omron, HBF-516B, Budapest, Hungary). After 5 min at rest both systolic and diastolic blood pressures were measured (Omron M10-IT).

#### Six-Minute Walking Test

Physical fitness was assessed by using a 6MWT, slightly different from the original protocol of Enright (2003). VO_2_max (L/min) was calculated as described by Laskin et al. (2007) and converted to relative VO_2_max (ml/kg/min).

#### Actigraph

Actigraph GT3X+ (Actigraph, Pensacola, FL, United States) is a tri-axial accelerometer-based method of monitoring activity (John and Freedson, 2012). Actigraph recorded movements in 60-s intervals. Participants were instructed to wear Actigraph continuously day and night for seven consecutive days at study entry, after 3- and 6-months of the program. Due to a limited number of Actigraphs, raw data collection was not performed on the exact same days for all participants, although all recordings were within the appropriate 1-week time period. Data were excluded from the analysis, if the participants did not wear the device for seven consecutive days. Actigraph recordings were analyzed using ActiLife 6.10 software (Actigraph) and cut-off values were calculated according to previous publications (Freedson et al., 1998).

#### Heart Rate Monitoring

R-R interval was registered every 2nd week during exercise sessions with a Polar Team System (Polar Electro Oy, Kempele, Finland) following Baynard’s method (Baynard et al., 2004). The transmitters were worn by the participants in ventral position under the tip of the xiphoid process in the middle of their chest.

Heart Rate monitoring was accomplished according to recommendations (Baynard et al., 2004). Briefly, subjects were first seated for 5 min to obtain resting HR. Then each subject completed the 30-min resistance exercise followed by the 20-min aerobic exercise with continuous HR monitoring. Finally, HR was continuously recorded for another 5 min in seated position to obtain recovery HR. Results were processed using Polar Pro Trainer 5, and Kubios HRV software.

#### Blood Sample Collection and Evaluation

Fasting blood samples were taken in the morning. Subjects were instructed to restrain from exercise during the day before their examination. Venous blood samples were collected in suitable vacutainers, tubes containing potassium ethylenediaminetetra-acetic acid (K-EDTA) were used for testing cellular blood parameters. Tubes without additives were used to obtain serum for C-reactive protein (CRP) and cortisol tests. After blood collection serum was separated by centrifugation (10 min, room temperature, 1500 rcf). Blood cell parameters were quantified in a multi-parameter automatic hematology analyzer Cell-Dyn 3700 system (Abbott Diagnostics, Abbott Laboratories, Abbott Park, IL, United States). CRP was measured by Cardiac C-Reactive Protein (Latex) High Sensitive turbidimetric immunoassay (Roche Diagnostics) on Cobas 8000 Modular Analyzer (Roche Diagnostics, GmbH, Mannheim, Germany) following the manufacturer’s instructions. Cortisol was measured by Cortisol RIA Kit (Beckman Coulter, Cat.: IM1841) on RIA-mat 280 automated analyzer (Stratec Gmbh, Birkenfeld, Germany) following the manufacturers’ instructions.

#### Quantification of hTrec Copy Number Using Digital PCR

Venous blood samples were collected in sodium citrate-containing tubes (BD Vacutainer Blood Collection Tube). Genomic DNA was isolated from 200 μl blood sample using Blood Mini Kit (Qiagen, Hilden, Germany), allowing for sjTRECs (also called hTREC) DNA loops to co-isolate with genomic DNA. Total DNA was quantified using NanoDrop 2000 (Thermo Fisher Scientific, United States). DNA was then diluted to a final concentration of 2000–3000 copies/μl. Digital PCR reactions were prepared according to the QuantStudio^TM^ 3D Digital PCR Reagent Kit (Thermo Fisher Scientific) recommendation. The Digital PCR sample mix was added on each chip and loaded on ProFlex^TM^ 2x Flat PCR System following the manufacturer’s instructions. Absolute quantification of hTrec copy number was performed using QuantStudio 3D Digital PCR System (Thermo Fisher Scientific) then analyzed using Analysis SuiteCloud Software.

#### Artificial Neural Network Correlation of Physiological and Molecular Data

Evaluation of the dataset was carried out by Neurosolutions 6 (Neurodimension Inc.) ANN software of Jordan/Elman type. As being a highly adaptive system, this kind of network extends the basic multilayer perceptron model with context units remembering previous. The network can reveal the hidden relationship between input and output factors of no obvious connection. For network training 14 datasets were used (lifestyle program participants) throughout 10 000 training sessions with an average mean squared error of 0.003. Software. Significance was determined using simple *t*-test.

#### Biostatistics Evaluation of Physiological and Molecular Data

Euclidean distance matrix was computed based on the acquired data. To detect any pattern in this dataset hierarchical clustering with complete agglomeration method was used. For key ANN parameters (sedentary status, insulin, hTREC) we created dendrograms based on clustering results. In addition to visualize data pattern, 3D plots were also employed using NCSS version 12 statistical software.

### Statistical Analyses

To analyze data absolute change was used to measure the difference from baseline since relative change, unlike suggested in literature (Vickers, 2001) prooved statistically inefficient. Then data were converted to Z-scores and shown as means ± SD. Numerous studies have shown that K-means cluster analysis reveals human responsiveness in an unbiased manner (Bamman et al., 2007). With the use of K-means cluster analysis (SPSS version 22 for windows) we have identified two clusters: low responders (LR) and high responders (HiR). Individuals showing biological improvement were termed HiR and individuals with negligible improvement or deterioration were termed LR. After the identification of the two groups (LR and HiR) these were tested by One-Way ANOVA using *p* < 0.05. Power analysis was also performed for confirmation (please refer to Supplementary Material S3).

## Results

### Physiological Parameters

**FIGURE 1:**
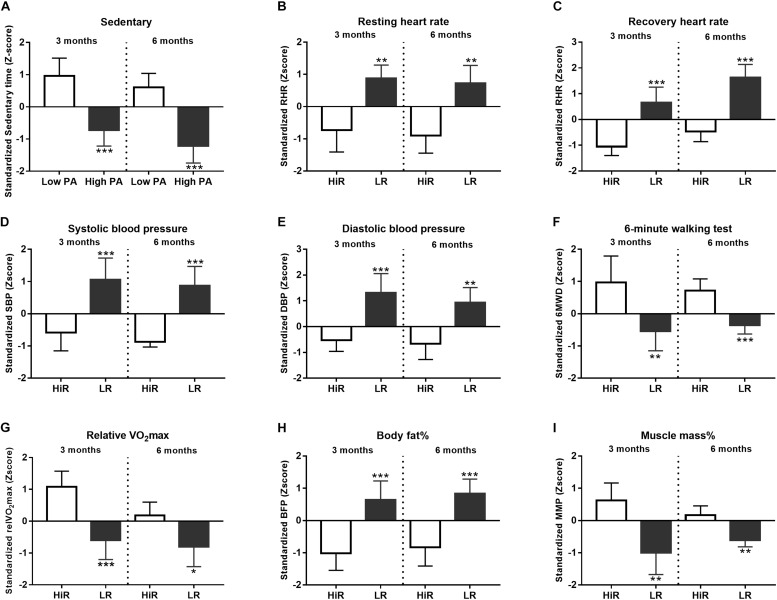
Individual variation of physiological parameters in response to lifestyle program. **(A–I)** K-means cluster analysis was used to differentiate high- (HR) and low (LR) responders (*n* = 14). Bar graph values indicate standardized Z-score values, and standard deviation values. 3- and 6-month values were normalized to baseline values and converted to Z-scores. ^∗^Significant difference between HR and LR (*p* < 0.05). ^∗∗^Significant difference between HR and LR (*p* < 0.01). ^∗∗∗^Significant difference between HR and LR (*p* < 0.001). Please refer to Supplementary Material S1 for exact numerical values.

Changes in classical physiological parameters occurring during a lifestyle program are well characterized (Galani and Schneider, 2007). Therefore, to validate our program we confirmed that young and healthy, but previously inactive individuals enrolled in our 6 month-long program show the anticipated gains in these physiological parameters. Figure 1 summarizes changes in such classical physiological parameters. K-means cluster analysis of absolute scores was performed for all the recorded parameters to differentiate HiR and LR followed by normalization to obtain half time (0–3 months) and final results (0–6 months). We focused on cluster analysis results to differentiate HiR and LR individuals. Exact numerical values of the recorded parameters are listed in Supplementary Material S1.

#### Physical Activity

Physical activity of the recruited participants was recorded and evaluated. By definition inactive individuals spend a significant amount of time in sedentary status, hence increasing PA means that these individuals spend less time in sedentary behavior (Kohl et al., 2012). Z-score values of sedentary status cluster analysis (Figure 1A) show that at half time (3 months, left part of Figure 1A) high PA (High PA) and low PA (Low PA) clusters segregate significantly (*p* < 0.001). Please, note that both clusters show increasing activity by the end of the program (6 months, right part of Figure 1A) without affecting the significance of cluster segregation (*p* < 0.001).

#### Cardiovascular System

Heart Rate- (Figures 1B,C) and blood pressure values (Figures 1D,E) were acquired and evaluated as direct cardiovascular parameters. Based on cluster analysis HiR and LR clusters segregate for resting- and recovery HR (Figures 1B,C). Resting HR Z-score remains low for HiR individuals during the program, but recovery HR Z-score shows mild increase in comparison of half time and final values. Meanwhile, the statistical power of segregation remains unchanged for both resting- (*p* < 0.01) and recovery- (*p* < 0.001) HR Z-score values. Blood pressure Z-score values obtained by cluster analysis (Figures 1D,E) also show the anticipated decrease due to regular PA in HiR individuals. In case of systolic blood pressure, the significance of HiR and LR segregation remains unaltered during the program (*p* < 0.001) (Figure 1D), while diastolic blood pressure Z-score values decrease in the significance of segregation for HiR and LR clusters (mid-term *p* < 0.001, final *p* < 0.01) (Figure 1E).

#### Physical Performance

The enrolled individuals completed a standard 6MWT at program entry, half time and exit. Cluster analysis identifies HiR and LR individuals at half time and program exit as well (Figure 1F). HiR individuals show improvement in the 6MWT. However, half time values after 3 months appear to outperform final values at 6 months as Z-score values decrease with time, even though the statistical power of cluster segregation increases (*p* < 0.01 at half time, and *p* < 0.001 at program exit) (Figure 1F). Since physical performance correlates with respiratory capacity, relative VO_2_max was also recorded and evaluated (Figure 1G). As expected VO_2_max increases in HiR individuals due to regular PA. Statistical analysis shows that HiR and LR individuals strongly segregate at half time (*p* < 0.001) showing slight decrease by the end of the program (*p* < 0.05). In support VO_2_max also shows a moderate decrease with time (Figure 1G), similar to the 6MWT results (Figure 1F) and the recovery HR (Figure 1C) Z-score values above.

#### Body Composition

Body-mass index (BMI) is calculated based on body mass and height. Regular PA is known to restructure body composition decreasing body fat content and increasing muscle mass (Said et al., 2017). However, since muscle density exceeds fat density BMI values can mask subtle changes of body composition. For this reason body fat percent and muscle mass percent were measured directly (Figures 1H,I). It is important to note that participants have not changed their dietary habits during the program. HiR and LR individuals segregate significantly according to cluster analysis. As anticipated, regular PA decreased body fat percent (Figure 1H) in an activity-proportional manner as HiR individuals lost fat (left part of Figure 1H), while LR individuals gained fat (right part of Figure 1H), with unaltered cluster segregation throughout the program (*p* < 0.001). Muscle mass percent shows the opposite tendency, albeit the effect is more profound for HiR individuals at half time (left part of Figure 1I) than at exit (right part of Figure 1I) despite unaltered significance of cluster segregation (*p* < 0.01).

### Molecular Parameters

Changes in classical molecular parameters were also anticipated to occur during a lifestyle program. This section focuses on cluster analysis to differentiate high responders (HiR) and low responders (LR) based on molecular parameters, while the exact numerical values are listed in Supplementary Material S1.

#### Glucose and Lipid Metabolism

**FIGURE 2:**
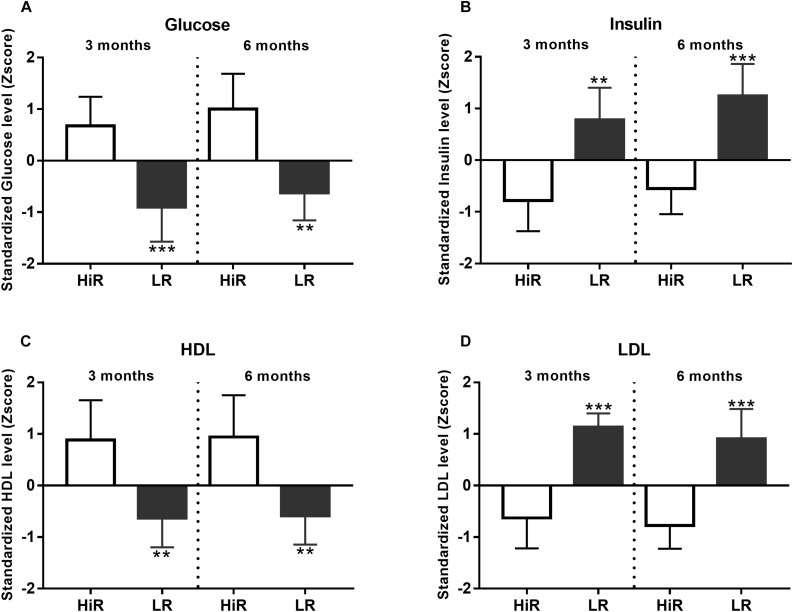
Individual variation of metabolic parameters in response to lifestyle program. **(A–D)** K-means cluster analysis was used to differentiate high- (HR) and low (LR) responders (*n* = 14). Bar graph values indicate standardized Z-score values, and standard deviation values. 3- and 6-month values were normalized to baseline values and converted to Z-scores. ^∗^Significant difference between HR and LR (*p* < 0.05). ^∗∗^Significant difference between HR and LR (*p* < 0.01). ^∗∗∗^Significant difference between HR and LR (*p* < 0.001). Please refer to Supplementary Material S1 for exact numerical values.

Regular PA is known to depend on and gradually reshape energy utilization, both fast-acting glucose and slow-acting lipid metabolism (Yu et al., 2016). For this reason basic relevant metabolic parameters have been recorded and evaluated using cluster analysis. Figures 2A,B show glucose and insulin level Z-scores value, at half time and program exit. HiR of glucose metabolism show moderate increase of fasting glucose level, while their insulin show a moderate decrease of fasting insulin level at half time and program exit as a steady-state adaptation to regular PA. Both parameters show significant segregation of HiR and LR individuals. Please, note that there is decreasing glucose and increasing insulin Z-score segregation with time (*p* < 0.001 to *p* < 0.01 for glucose, and *p* < 0.01 to *p* < 0.001 for insulin, comparing half time and final values, respectively). As for lipid metabolism, HDL and LDL cluster analysis results are shown by Figures 2C,D. As expected, regular PA increases HDL, but decreases LDL values in HiR individuals. Unlike glucose metabolism above, HiR and LR segregation does not change during the program (*p* < 0.01 for HDL and *p* < 0.001 for LDL).

#### Immune System

Regular PA is considered to enhance immune responses due to chronic low-level stress also known as hormesis (Pedersen and Hoffman-Goetz, 2000). Since hormesis enhances stress resistance partly due to increased endogenous steroid-response, cortisol level was also recorded (Budde et al., 2015). In order to identify HiR and LR individuals in terms of the immune system, basic parameters including an acute phase protein (CRP) and lymphocyte counts were also evaluated. The endocrine system (via e.g., cortisol) is known to affect both the metabolic system (represented above by glucose and insulin levels) and the immune system (represented here by lymphocyte counts) (Hackney and Lane, 2015).

**FIGURE 3:**
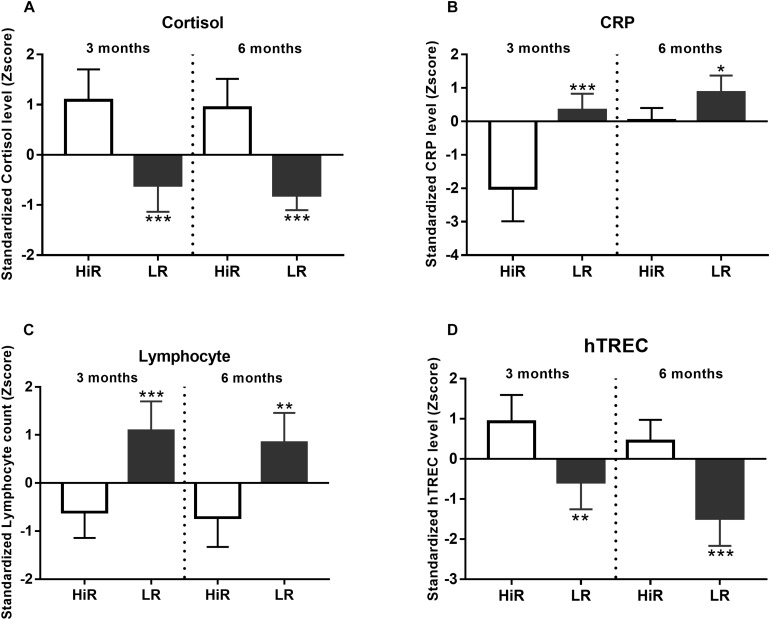
Individual variation of immunological parameters in response to lifestyle program. **(A–D)** K-means cluster analysis was used to differentiate high- (HR) and low (LR) responders (*n* = 14). Bar graph values indicate standardized Z-score values, and standard deviation values. 3- and 6-month values were normalized to baseline values and converted to Z-scores. ^∗^Significant difference between HR and LR (*p* < 0.05). ^∗∗^Significant difference between HR and LR (*p* < 0.01). ^∗∗∗^Significant difference between HR and LR (*p* < 0.001). Please refer to Supplementary Material S1 for exact numerical values.

Cortisol levels increase following regular exercise in HiR individuals, and cluster analysis shows marked segregation of HiR and LR (Figure 3A, *p* < 0.001) both at half time and program exit. CRP level Z-score values show the anticipated decrease in HiR individuals and a marked segregation of HiR and LR individuals at half time (Figure 3B, *p* < 0.001) that unexpectedly diminishes by program exit (at 6 months *p* < 0.05). Elevated cortisol levels are known to suppress specific leukocyte counts e.g., lymphocyte counts (Pedersen and Hoffman-Goetz, 2000). This is potentially supported by Figure 3C as lymphocyte counts decrease in HiR individuals. Cluster analysis shows marked and significant segregation of HiR and LR at both half time and program exit for lymphocyte counts (Figure 3C, *p* < 0.001 at mid-term and *p* < 0.01 at program exit). Thymus function (fresh naïve T-cell production) has been reported to improve with regular moderate physical exercise (Duggal et al., 2018). For this reason we have performed digital qPCR measurement to evaluate fresh naïve T-cell production via hTREC measurement (Hazenberg et al., 2001). hTREC is a DNA loop by-product of TcR (T-cell receptor) gene rearrangement produced during thymocyte maturation in the thymus. hTREC co-isolates with genomic DNA during isolation. Its level is proportional with thymus function: T-cell production. As anticipated, Z-scores in HiR individuals increased due to PA by half time (*p* < 0.01) that lessened by program exit (*p* < 0.001). Our data support that moderate regular exercise enhances thymus function (fresh naïve T-cell production).

### Correlation of Physiological and Molecular Parameters Using ANN

**FIGURE 4:**
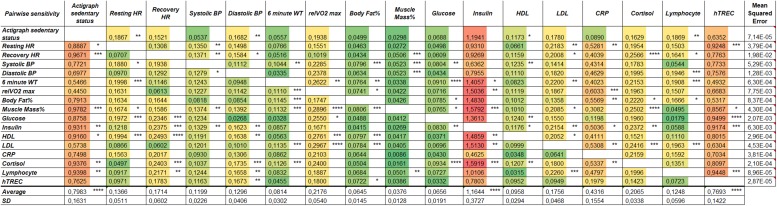
Artificial neural network (ANN) analysis of physiological and molecular parameters in response to lifestyle program. Heat map shows pairwise correlation efficiencies of physiological and molecular parameters (*n* = 14). Red color shows high correlation, green color indicates low correlation efficiency between parameters. Significance level of pairwise sensitivity correlation is shown by asterisks (^∗^*p* < 0.05, ^∗∗^*p* < 0.01, ^∗∗∗^*p* < 0.001, and ^∗∗∗∗^*p* < 0.0001). Significance was determined using simple *t*-test. Mean squared error in last column indicates model accuracy.

Our lifestyle program yielded a substantial dataset including physiological and molecular parameters. Concept-free ANN analysis was used to find specific correlation patterns among these parameters in a pairwise manner. Figure 4 shows pairwise correlation efficiency values visualized with a heat map. Physiological and molecular parameters with strong correlation efficiency received high marks (shown by red color), while weak correlation efficiencies received low marks (shown by green color). According to ANN analysis a physiological parameter (activity or sedentary status), a molecular metabolic parameter (insulin concentration) and a molecular immunological parameter (hTREC copy number) showed the strongest correlation with the rest of the recorded parameters appointing them as the most influential mastermind hubs of the current lifestyle program (Figure 4).

### Evaluation of Physiological and Molecular Parameters Using Biostatistics

**FIGURE 5:**
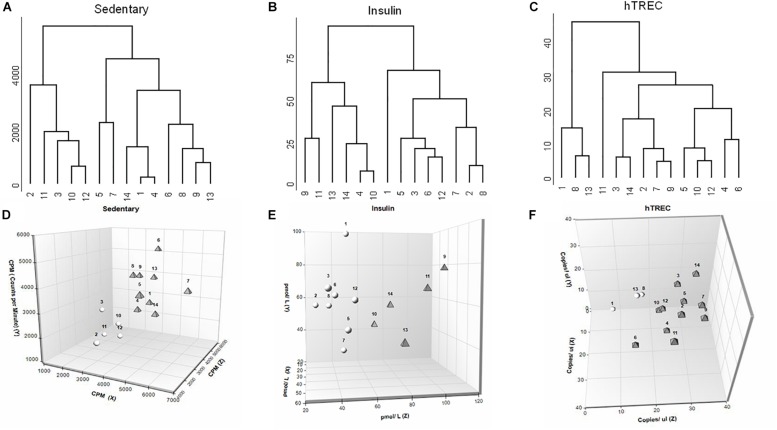
Biostatistics evaluation of physiological and molecular parameters in response to lifestyle program. **(A–C)** Dendrogram representation shows segregation of participants (*n* = 14) for sedentary status, insulin and hTREC. Please note that all three parameters show an early bifurcation indicating segregation into HR and LR individuals. **(D–F)** 3D plots allow for enhanced visualization of sedentary, insulin and hTREC parameter segregation. Please note the separation of HR and LR individuals (*n* = 14) represented by white spheres and gray triangles (view of angle was selected to aid the visualization of separation). *X*-axis shows baseline (0 month), *Y*-axis shows mid-term (3 months) and *Z*-axis shows program exit (6 months) values.

Artificial neural network analysis above appointed sedentary status, insulin and hTREC as the most influential, and deregulated parameters. We applied further analysis for these parameters in order to examine the segregation of participants. We have created a concept-free hierarchical clustering with complete agglomeration. Figure 5 shows the dendrogram representation of participants for sedentary status (Figure 5A), insulin (Figure 5B), and hTREC (Figure 5C). Please, note that all three parameters show an early bifurcation indicating the segregation of low- and high-responders as suggested by K-means statistics. We also show 3D plots for enhanced visualization of sedentary status (Figure 5D), insulin concentration (Figure 5E) and hTREC copy number (Figure 5F) segregation. Please, note the separation of low- and high responder individuals represented by white spheres and gray triangles (view of angle was selected to aid the visualization of separation). *X*-axis shows baseline (0 month), *Y*-axis shows half time (3 months), and *Z*-axis shows program exit (6 months) values. 2D plots are available for all the assessed physiological and molecular parameters in Supplementary Material S2.

## Discussion

### Physiological System

Physical activity proves to be the major physiological driving force that shows intimate connection with key metabolic (e.g., insulin concentration) and immune (e.g., hTREC copy number) parameters as confirmed by ANN analysis (Figure 4). This is in harmony with our working hypothesis suggesting that our lifestyle program achieves molecular level improvement through PA. The degree of PA significantly depends on personal compliance of the participants. Several lifestyle studies report amelioration after 3 months, as also observed in our program (Weston et al., 2014). However, long-term follow-up is advised, as changes in lifestyle are only effective if pursued over an extended period of time. If the major driving force (PA) weakens over time, this may also be reflected by target parameters. In harmony, although 6MWT results showed obvious and significant gain at half time, it diminished by program exit (Figure 1F). In support, recovery HR decent (Figure 1C) decelerates, VO_2_max gain diminishes (Figure 1G) and muscle mass percent gain fades (Figure 1I) at program exit.

### Metabolic System

Cortisol, a family member of corticosteroids, is also acknowledged as a hormone of adaptation that aids acclimatization to chronic low level physical stress triggered by regular exercise also called hormesis (Hackney and Lane, 2015). Cortisol levels showed significant increase by half time that remained sustained during the program in support of aerobic fitness improvement and the overall adaptation process (Figure 3A). Regular physical exercise provokes a tight glucose control, but not necessarily a lowered fasting glucose level. Along with elevated cortisol level this resulted in an indicative (not significant) increase of fasting blood glucose level, most noticeable in HiR individuals by program exit (Figure 2A). As expected based on glucose physiology, fasting insulin levels showed an anti-parallel, indicative decrease (Figure 2B). Regular physical exercise is also known to up-regulate the level of HDL cholesterol. Please, note the indicative changes of HDL level (Figure 2C) that show alignment with the extent of PA (Figure 1A). The recorded anti-parallel changes of HDL and LDL cholesterol levels are in harmony with literature data (Hagner-Derengowska et al., 2015).

### Immune System

Corticosteroids are potent anti-inflammatory agents (Pedersen and Hoffman-Goetz, 2000; Hackney and Lane, 2015). In our study CRP levels decrease during the program in response to increasing cortisol levels supporting the immune-biology relevance of enhanced cortisol production in the current lifestyle program setting (Figure 3B), although the effect is temporary as CRP decrease diminishes in HiR individuals by program exit. Cortisol is also known to suppress multiple pathways of cell activation in several leukocyte subtypes (Hackney and Lane, 2015), a direct target group being the lymphocyte population (Pedersen and Hoffman-Goetz, 2000). In harmony with cortisol level changes lymphocyte counts showed significant decrease in HiR vs. LR individuals (Figure 3C). T-cells developing in the thymus (termed thymocytes) are especially sensitive to cortisol and readily undergo apoptosis (Sajdel-Sulkowska et al., 1978). However, thymus function (naïve T-cell production) has been reported to show improvement in response to moderate regular exercise (Duggal et al., 2018). In order to detect the net effect of cortisol (suppressing T-cell production) and regular exercise (improving T-cell production) we have measured the quantity of a T-cell production by-product (hTREC or human T-cell receptor excision circle) in peripheral blood lymphocytes. As anticipated, hTREC values showed significant increase by half time of the program, emphasizing the net immunological gain achieved by regular moderate exercise (Figure 3D).

### Personal Responsiveness

It has been reported that personal responsiveness relies on multiple factors, including genetics and environmental factors (physical and mental stress, sleep quality, dietary habits etc.) (Mann et al., 2014). Fixed training programs not aligned with personal requirements yield significant standard deviation, whereas personalized training sessions provide more coherent results (Mann et al., 2014). For this reason subjects enrolled in the current program performed moderate, regular exercise under the guidance of a personal trainer. Despite all possible precautions participating individuals segregate into high-responder (HiR) and low-responder (LR) individuals for all the assessed parameters. Of extreme importance HiR individuals of a particular parameter may prove to be LR individuals of another parameter. The phenomenon is easily observed by comparing the biostatistics dendrograms of our key ANN parameters (sedentary status, insulin, hTREC) as shown by Figures 5A–C. Please, note individual responsiveness patterns showing significant person-to-person variation. Our results highlight the diversity of human healthy young population in terms of responsiveness observed during combined endurance and strength-training program. A responsiveness table (please refer to Supplementary Material S4) provides further detail for all the assessed parameters in every participant, separately.

## Conclusion

Our observations provide novel information on individual differences in response to combined endurance and resistance training (Hautala et al., 2006). Previous studies mainly focused on individual response patterns of endurance training alone (Scharhag-Rosenberger et al., 2012; Mann et al., 2014). In most studies the frequency and intensity of training sessions was pre-determined and did not allow positive adaptive changes for the individuals and to fully recover from each session (Bouchard and Rankinen, 2001; Vollaard et al., 2009; Scharhag-Rosenberger et al., 2012). In contrast to such fixed training sessions in our study participants performed moderate, regular exercise individually adjusted to HR monitoring results during the 6 months under the guidance of a personal trainer. Such personalization suits individual needs and abilities, and improves the coherence of results. Our study showed considerable variation in training adaptation for parameters like relVO2max, blood pressure, glucose, insulin, HDL, LDL, and cortisol, all in healthy and young, previously inactive individuals. More importantly, similarly to other studies, our data confirm that high responders for one parameter are not necessarily high responders for others (Vollaard et al., 2009; Mann et al., 2014). Apparently, the more parameters are assessed, the more individual health-improvement patterns are revealed. Such individual patterns of thymic naïve T cells production (through hTrec) are of considerable interest, since immune-senescence is a major trigger of senior frailty causing elevated incidence of infection, cancer and autoimmunity. To our knowledge in the present study we have shown for the first time the correlation of hTrec and regular PA.

A frequent misconception is to generalize data and present mean response alone, however, individual responses typically show remarkable variation, including segregation of high and low responders for each and every parameter. Using concept-free techniques, performing ANN analysis and biostatistics evaluation we have identified numerous molecular changes triggered by regular PA, and show evidence for individual responsiveness at molecular level. Our study proposes that ANN analysis combined with biostatistics evaluation – especially hierarchical cluster analysis – will help in the future to predict individual molecular responsiveness, however, more participants and further studies are required for proof-of-concept.

## Ethics Statement

The program was approved by the ethics committee of the University of Pécs (ref. no.: 6439/2016). All participants gave written informed consent before starting the lifestyle program in accordance with the Declaration of Helsinki. Experiments involving human subjects and their blood samples were performed with the consent of the Regional and Local Ethics Committee of Clinical Centre, University or Pécs according to their guidelines.

## Author Contributions

KG performed many anthropometric and most molecular measurements, and involved in the manuscript preparation. ZA performed the statistical evaluation of the assessed parameters. RH executed biostatistics evaluation of the assessed parameters. TN designed and orchestrated, while EK executed most physiological measurements. SP performed the ANN analysis. AG planned and supervised the biostatistics evaluation. JP involved in trial design of molecular procedures and manuscript preparation. MW involved in anthropometric and physiological trial design, and manuscript preparation. KK orchestrated the current research, involved in the design of most molecular procedures, and took significant part in the manuscript preparation.

## Conflict of Interest

The authors declare that the research was conducted in the absence of any commercial or financial relationships that could be construed as a potential conflict of interest. The reviewer IB-M declared a shared affiliation with the authors and a past co-authorship with one of the authors RH to the handling Editor.
