# Supplementary material for: Artificial Neural Network Correlation and Biostatistics Evaluation of Physiological and Molecular Parameters in Healthy Young Individuals Performing Regular Exercise
Source: Front Physiol. 2019 Oct 2;10:1242. doi: 10.3389/fphys.2019.01242 (PMC6797842; doi:10.3389/fphys.2019.01242)
Supplement: Supplementary file 3 [file Data_Sheet_3.docx]

**Sample size calculation:**

**F tests -** ANOVA: Fixed effects, omnibus, one-way

**Analysis:** A priori: Compute required sample size

**Input:** Effect size f = 0.8352444

α err prob = 0.05

Power (1-β err prob) = 0.80

Number of groups = 2

**Output:** Noncentrality parameter λ = 9.7668649

Critical F = 4.7472253

Numerator df = 1

Denominator df = 12

Total sample size = 14

Actual power = 0.8179946

**The actual p-values with relevant effect size:**

|  | **Actual p value between HR and LR** | **Effect size** | **Power (1-β)** |
| --- | --- | --- | --- |
| Sedentary status03 | p=0.000 | 0.751 | 1.000 |
| Sedentary status06 | p=0.000 | 0.803 | 1.000 |
| Resting HR03 | p=0.001 | 0.702 | 0.983 |
| Resting HR06 | p=0.003 | 0.734 | 0.962 |
| RecoveryHR03 | p=0.000 | 0.751 | 0.999 |
| RecoveryHR06 | p=0.000 | 0.85 | 1.000 |
| Systolic BP03 | p=0.000 | 0.662 | 0.993 |
| Systolic BP06 | p=0.000 | 0.826 | 1.000 |
| Diastolic BP03 | p=0.000 | 0.742 | 1.000 |
| Diastolic BP06 | p=0.001 | 0.673 | 0.983 |
| 6-minuteWT03 | p=0.002 | 0.560 | 0.947 |
| 6-minuteWT06 | p=0.000 | 0.766 | 0.998 |
| relVO2max03 | p=0.000 | 0.702 | 0.998 |
| relVO2max06 | p=0.018 | 0.521 | 0.734 |
| BodyFat%03 | p=0.000 | 0.704 | 0.996 |
| BodyFat%06 | p=0.000 | 0.753 | 0.753 |
| MuscleMass%03 | p=0.001 | 0.678 | 0.992 |
| MuscleMass%06 | p=0.001 | 0.754 | 0.996 |
| Glucose03 | p=0.000 | 0.656 | 0.992 |
| Glucose06 | p=0.001 | 0.68 | 0.992 |
| Insulin03 | p=0.001 | 0.667 | 0.98 |
| Insulin06 | p=0.000 | 0.741 | 0.999 |
| HDL03 | p=0.003 | 0.608 | 0.942 |
| HDL06 | p=0.002 | 0.599 | 0.958 |
| LDL03 | p=0.000 | 0.764 | 1.000 |
| LDL06 | p=0.000 | 0.758 | 1.000 |
| CRP03 | P=0.000 | 0.717 | 0.999 |
| CRP06 | p=0.011 | 0.491 | 0.799 |
| Cortisol03 | p=0.000 | 0.709 | 0.999 |
| Cortisol06 | p=0.000 | 0.816 | 1.000 |
| Lymphocyte03 | p=0.000 | 0.706 | 0.998 |
| Lymphocyte06 | p=0.001 | 0.653 | 0.985 |
| hTREC03 | p=0.002 | 0.585 | 0.947 |
| hTREC06 | p=0.000 | 0.712 | 0.997 |
