# Supplementary material for: Artificial Neural Network Correlation and Biostatistics Evaluation of Physiological and Molecular Parameters in Healthy Young Individuals Performing Regular Exercise
Source: Front Physiol. 2019 Oct 2;10:1242. doi: 10.3389/fphys.2019.01242 (PMC6797842; doi:10.3389/fphys.2019.01242)
Supplement: Supplementary file 4 [file Data_Sheet_4.docx]

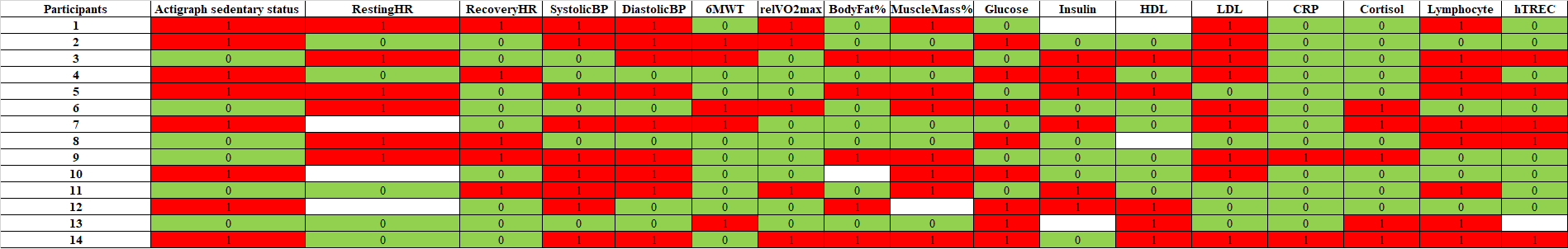


Legend of supplementary material (responsiveness table): Heat map classifies participants based on individual responsiveness for physiological and molecular parameters. Red color shows individuals with above average response (high responder or HR), green color indicates individuals with below average response (low responder or LR) while x indicates missing value.
