# Supplementary material for: Artificial Neural Network Correlation and Biostatistics Evaluation of Physiological and Molecular Parameters in Healthy Young Individuals Performing Regular Exercise
Source: Front Physiol. 2019 Oct 2;10:1242. doi: 10.3389/fphys.2019.01242 (PMC6797842; doi:10.3389/fphys.2019.01242)

Figure legend: Distribution of Z-score values after 6 months of regular exercise.

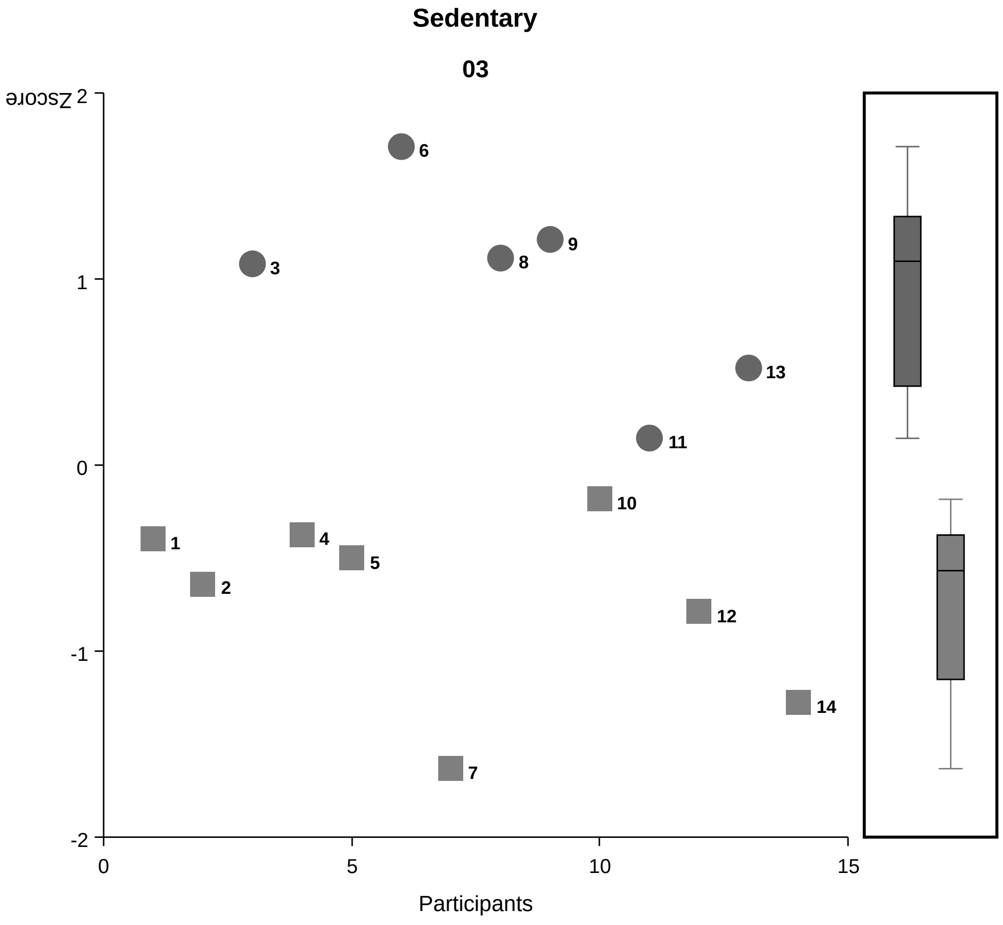

Figure legend: Distribution of Z-score values after 6 months of regular exercise.

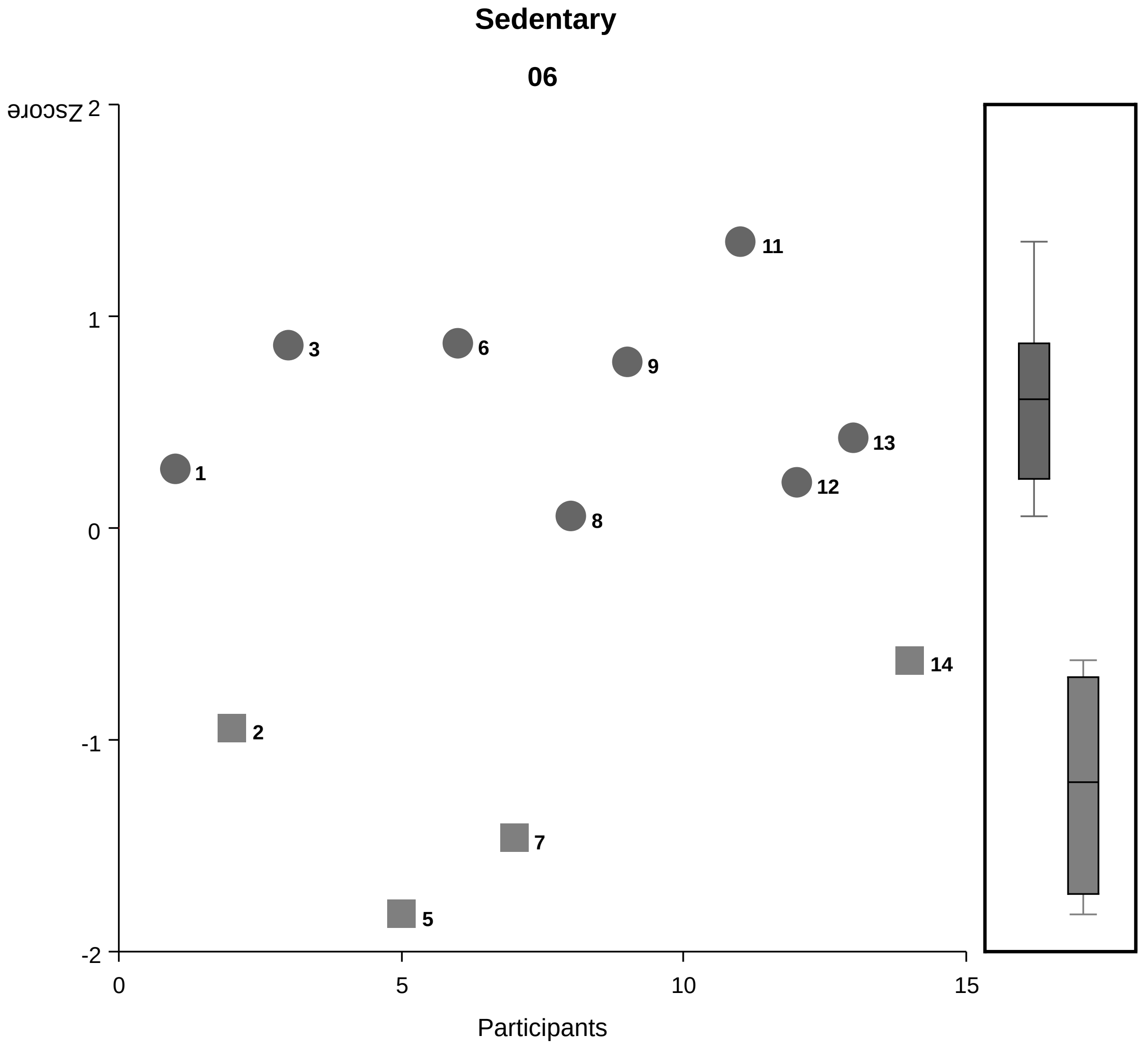

Figure legend: Distribution of Z-score values after 6 months of regular exercise.

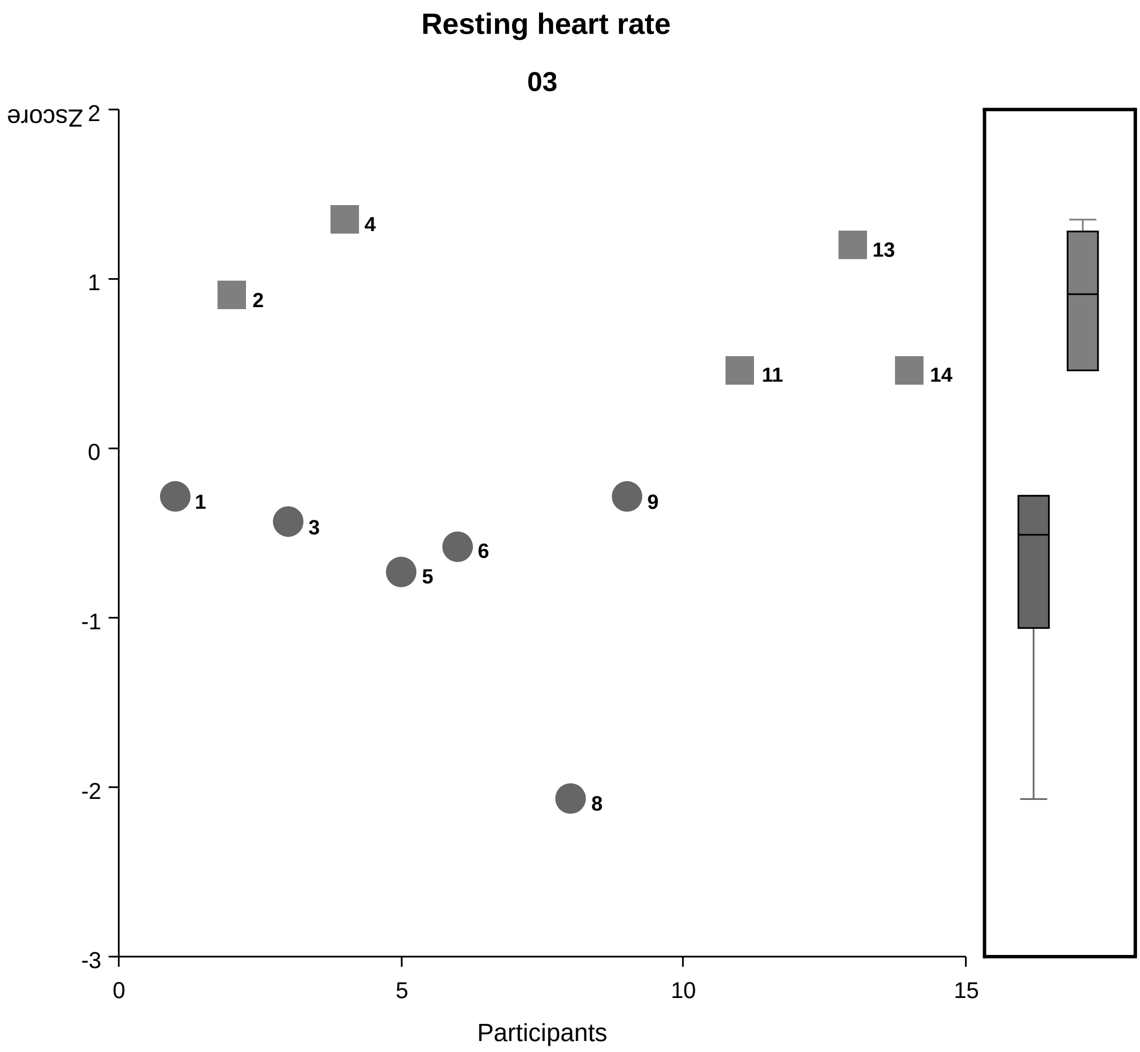

Figure legend: Distribution of Z-score values after 6 months of regular exercise.

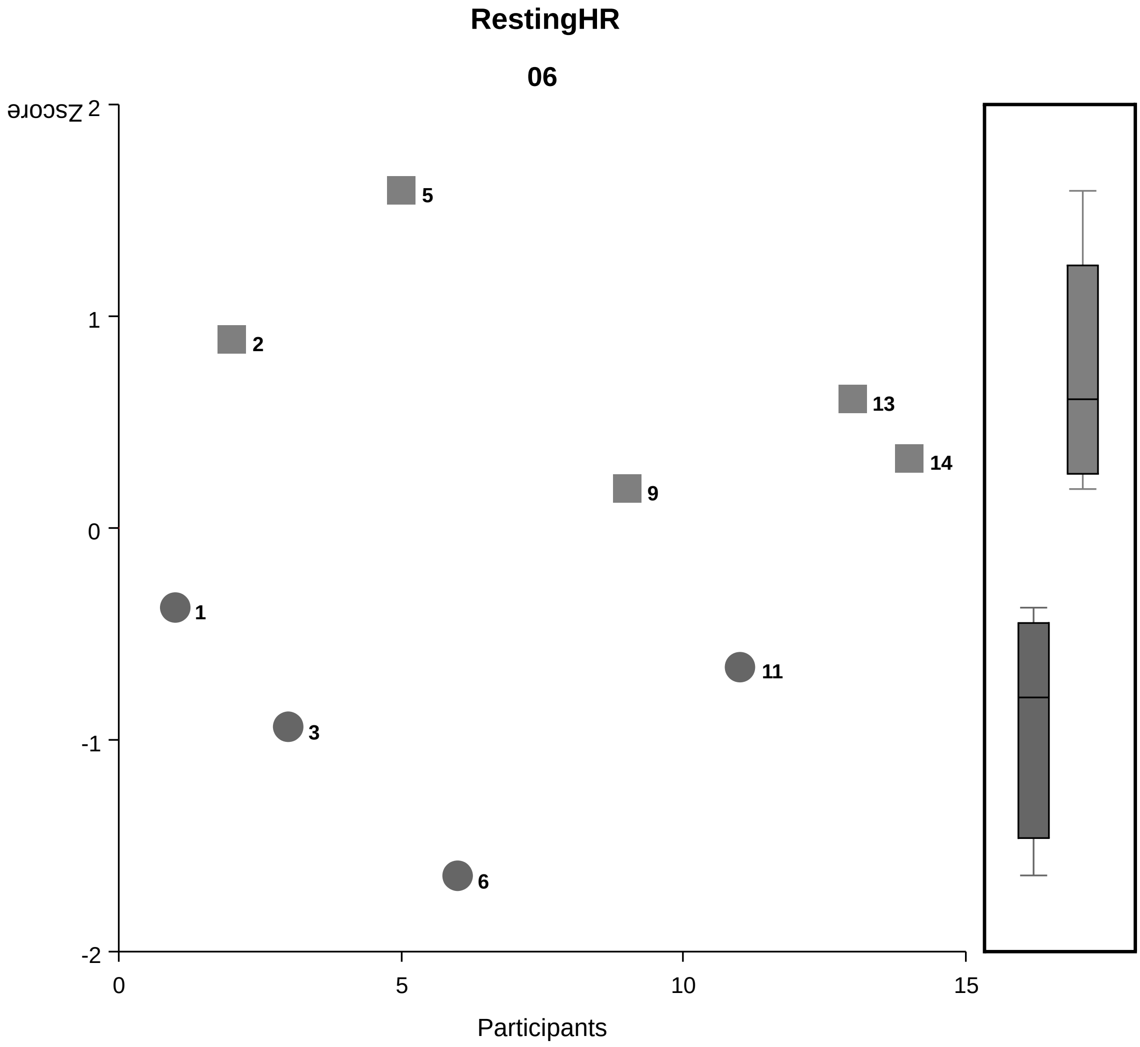

Figure legend: Distribution of Z-score values after 6 months of regular exercise.

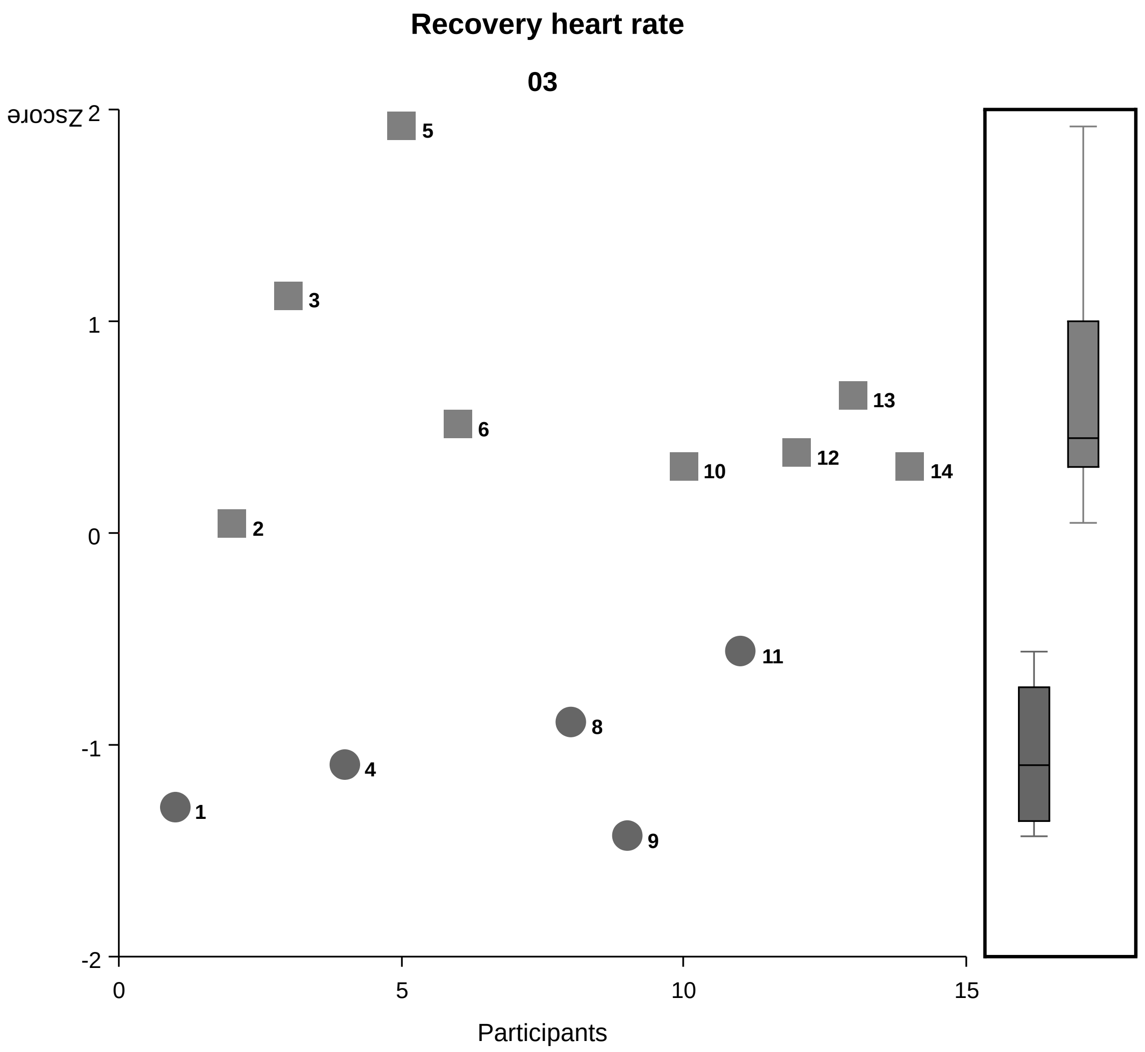

Figure legend: Distribution of Z-score values after 6 months of regular exercise.

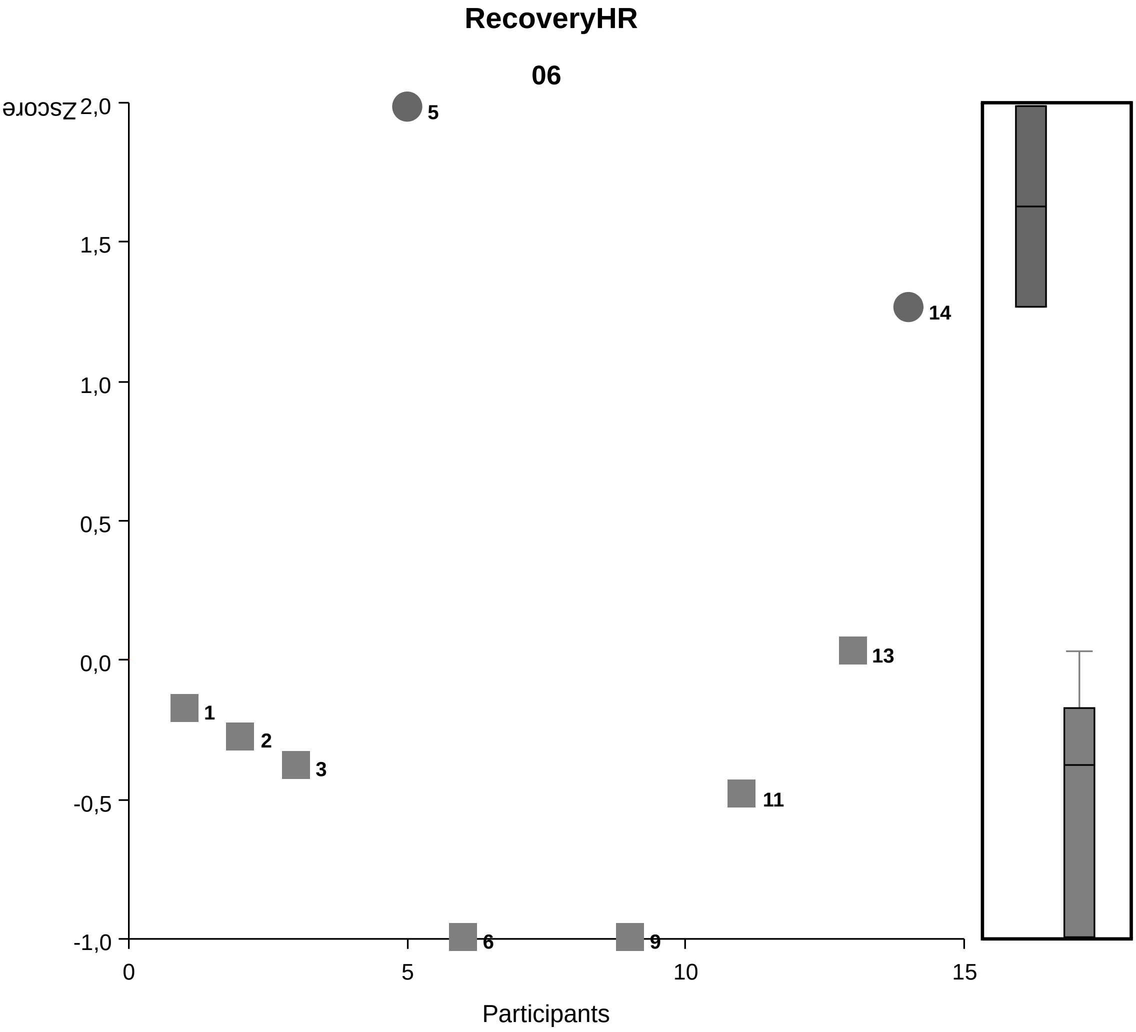

Figure legend: Distribution of Z-score values after 6 months of regular exercise.

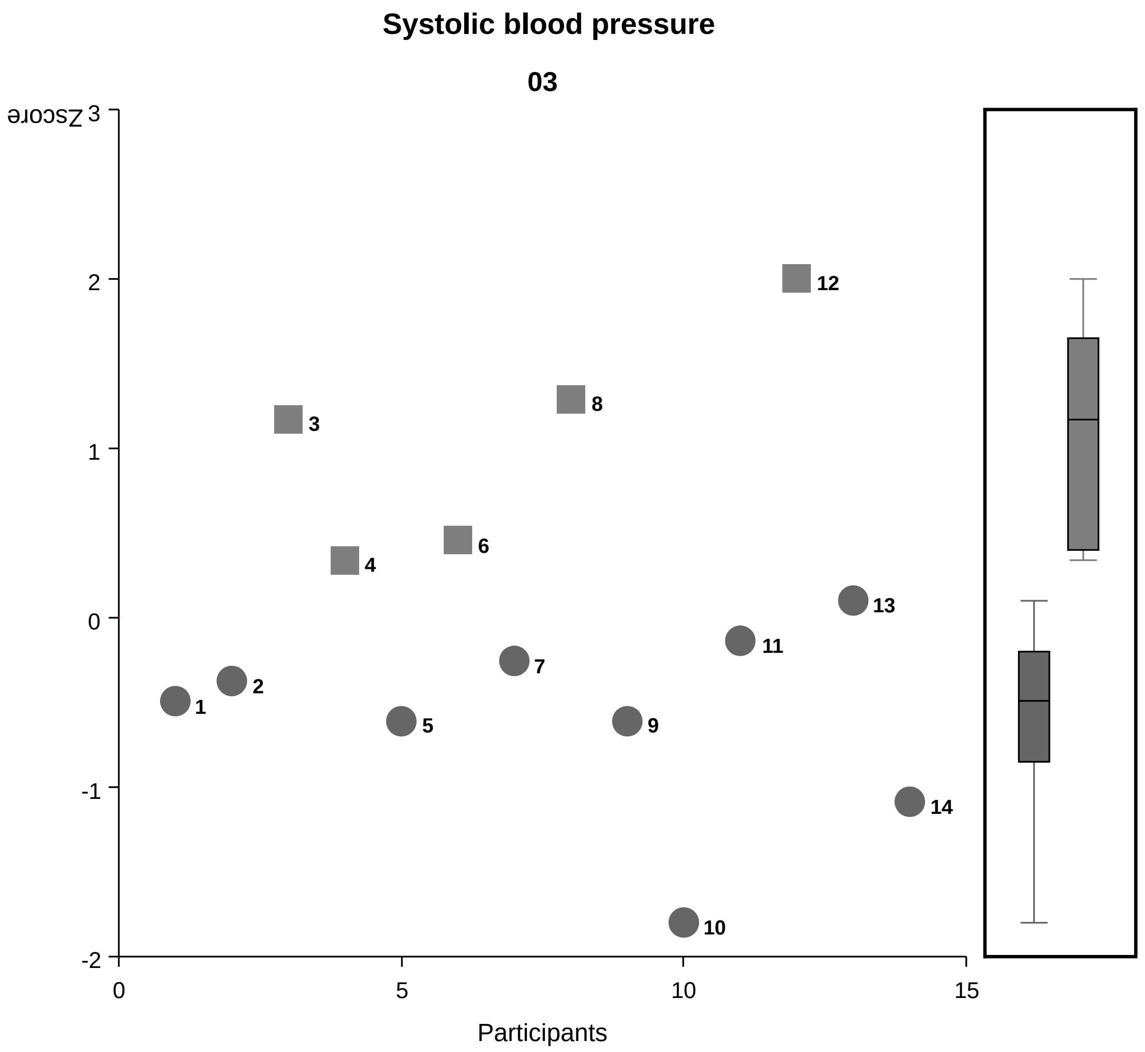

Figure legend: Distribution of Z-score values after 6 months of regular exercise.

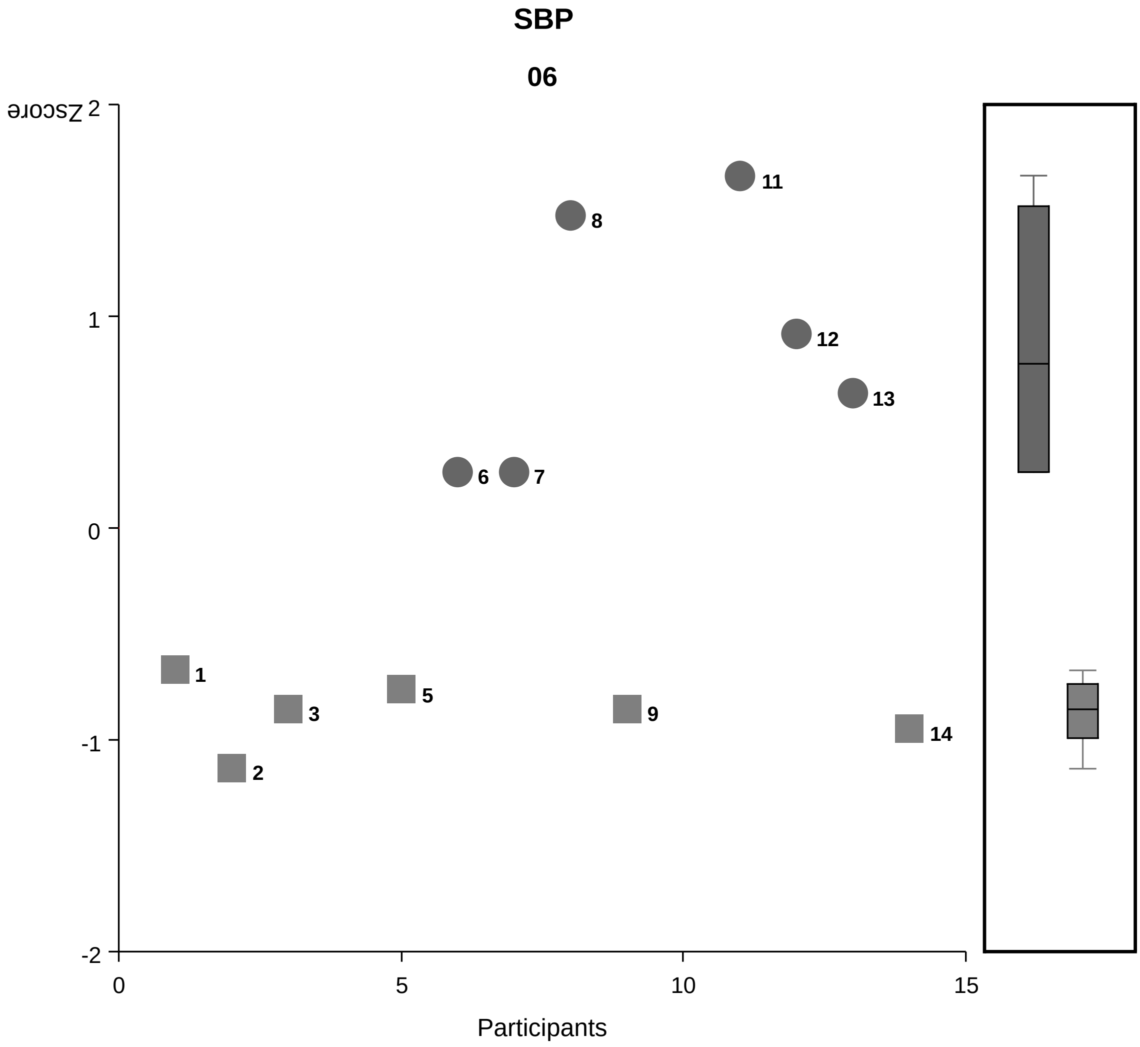

Figure legend: Distribution of Z-score values after 6 months of regular exercise.

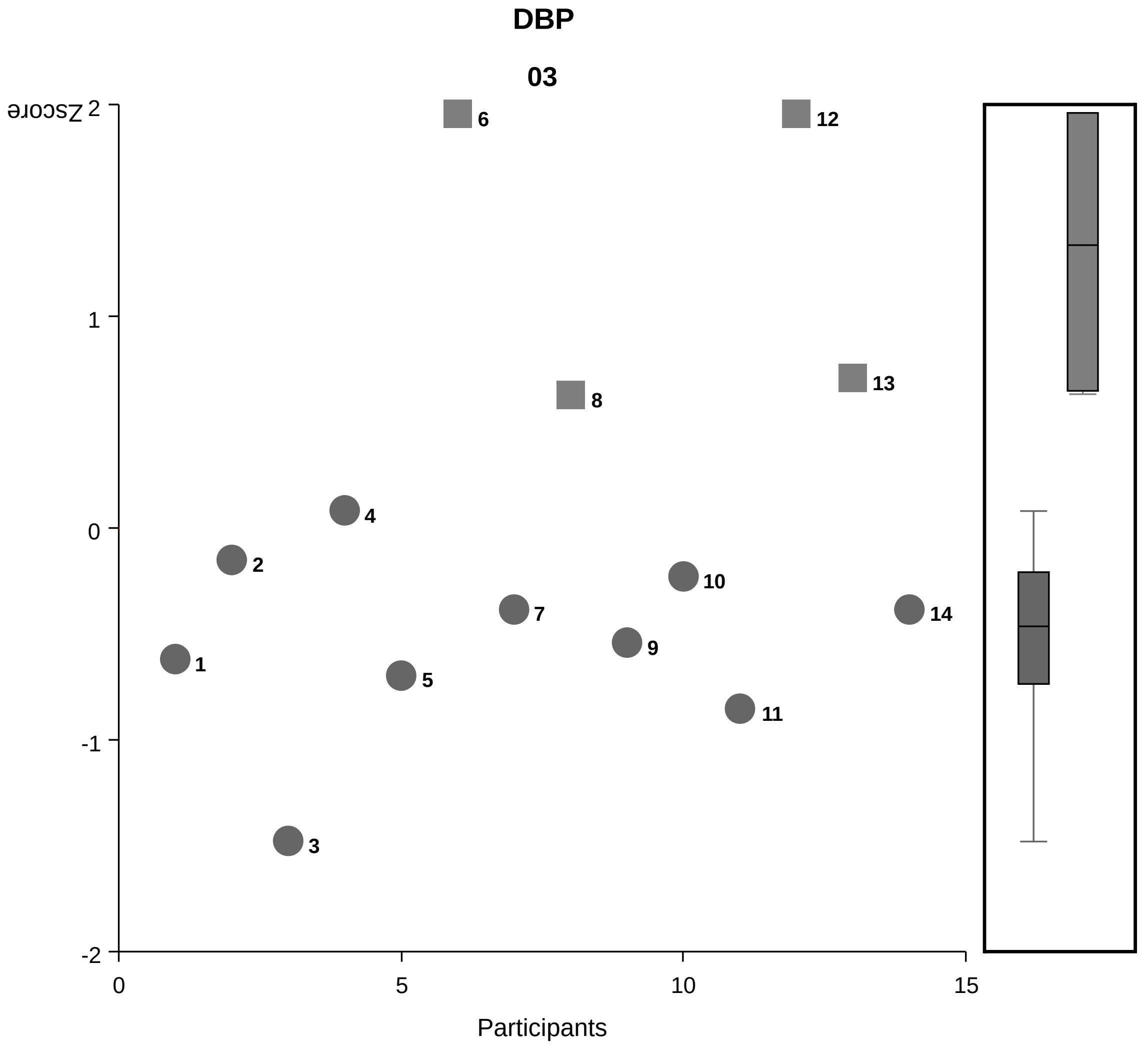

Figure legend: Distribution of Z-score values after 6 months of regular exercise.

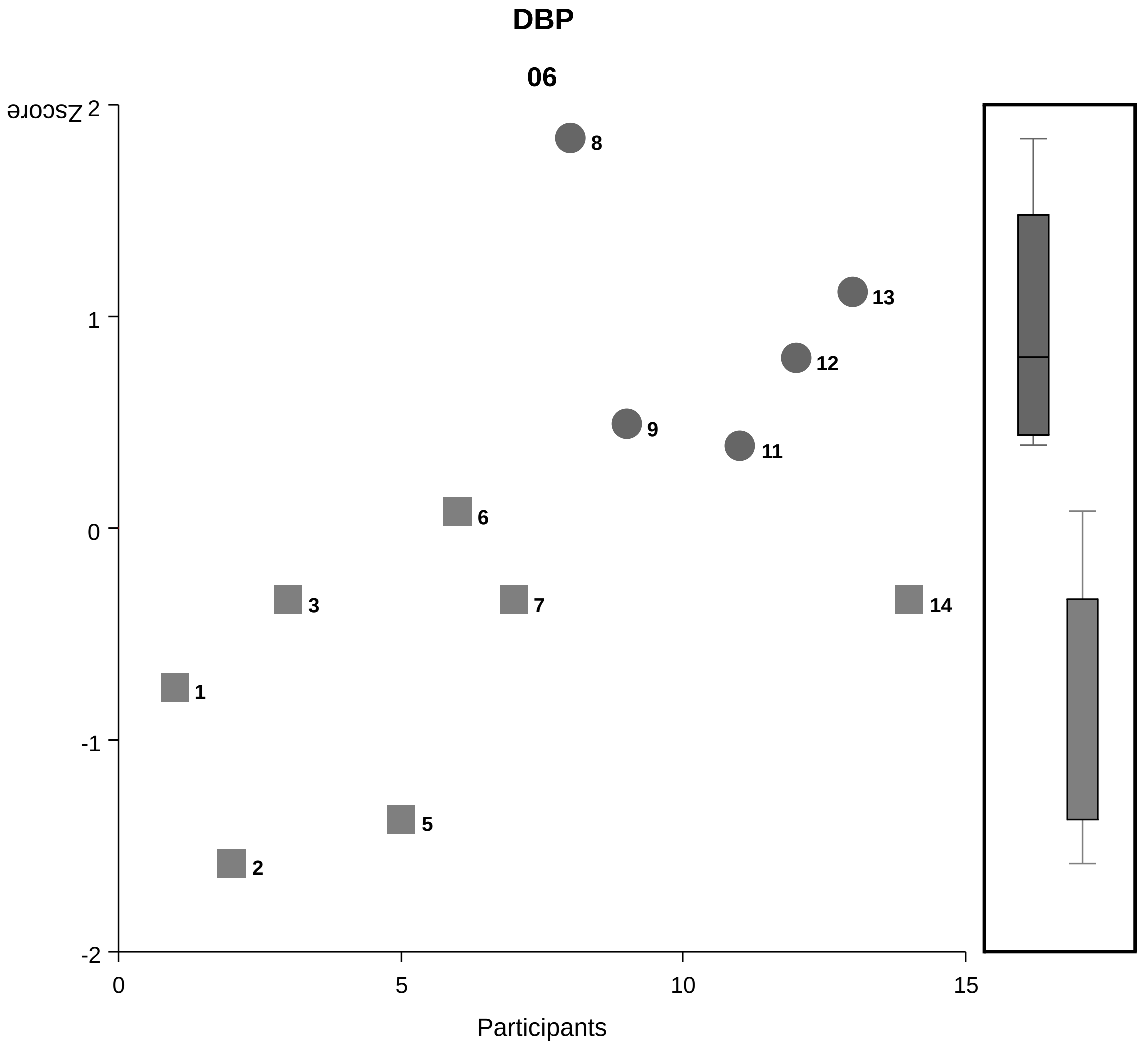

Figure legend: Distribution of Z-score values after 6 months of regular exercise.

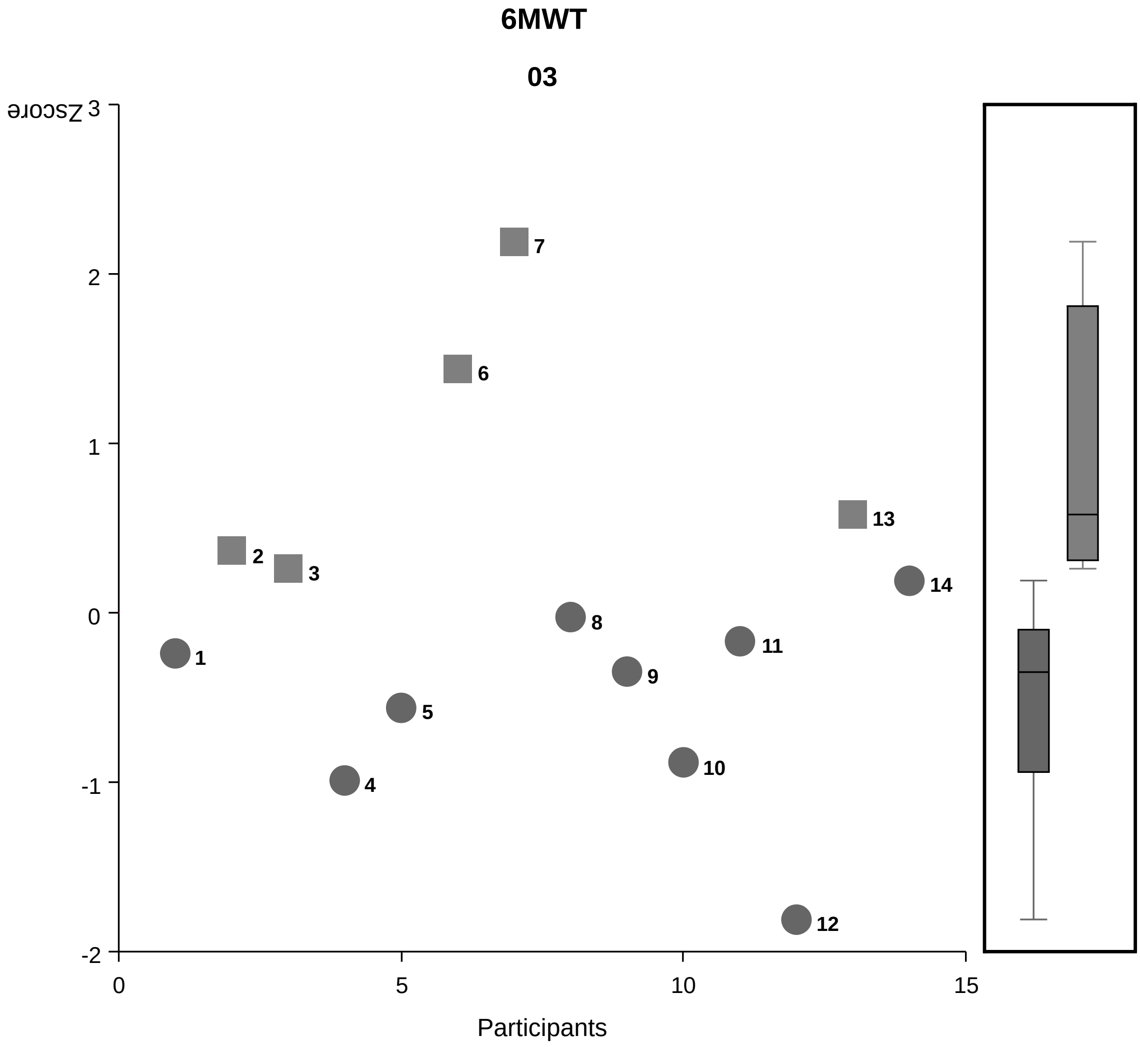

Figure legend: Distribution of Z-score values after 6 months of regular exercise.

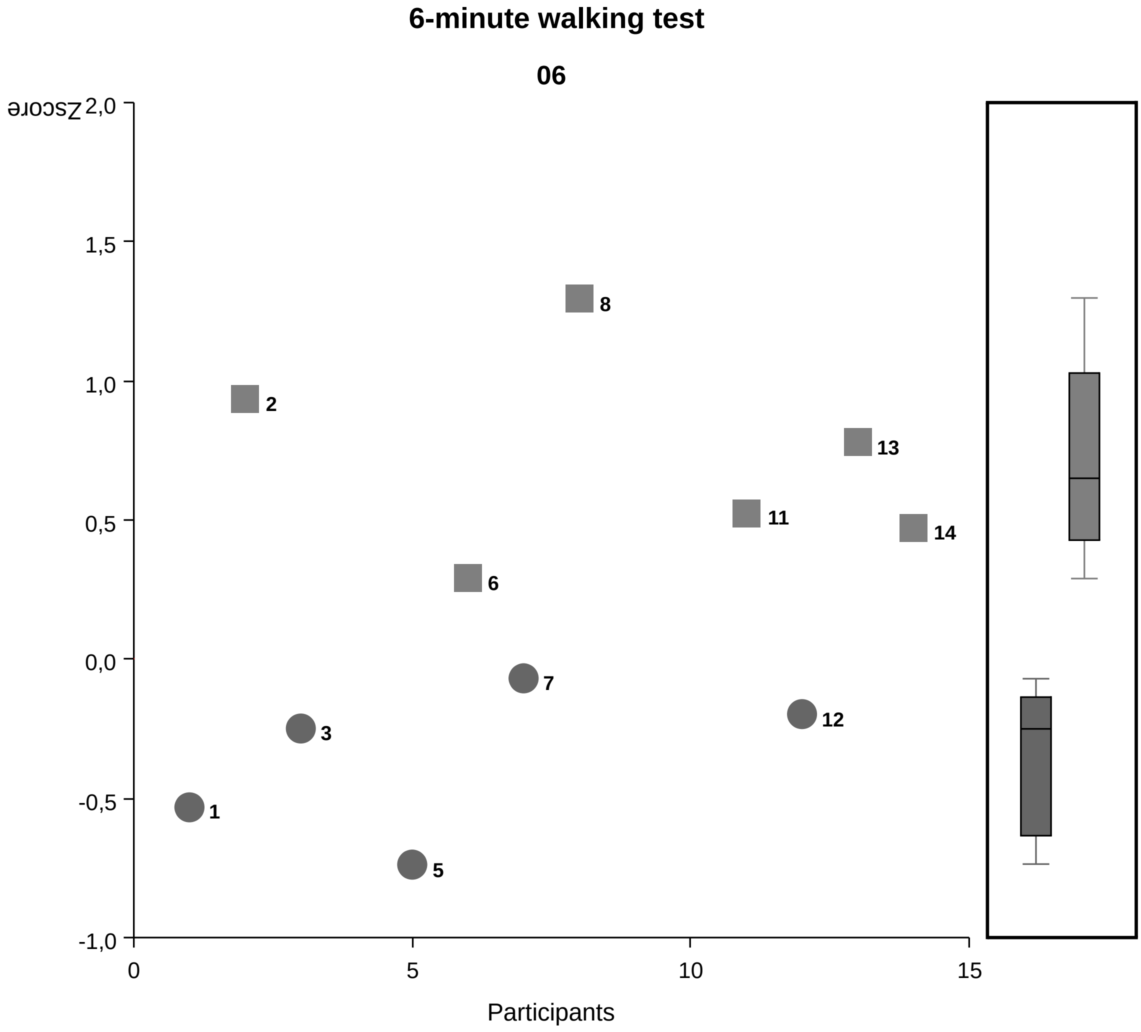

Figure legend: Distribution of Z-score values after 6 months of regular exercise.

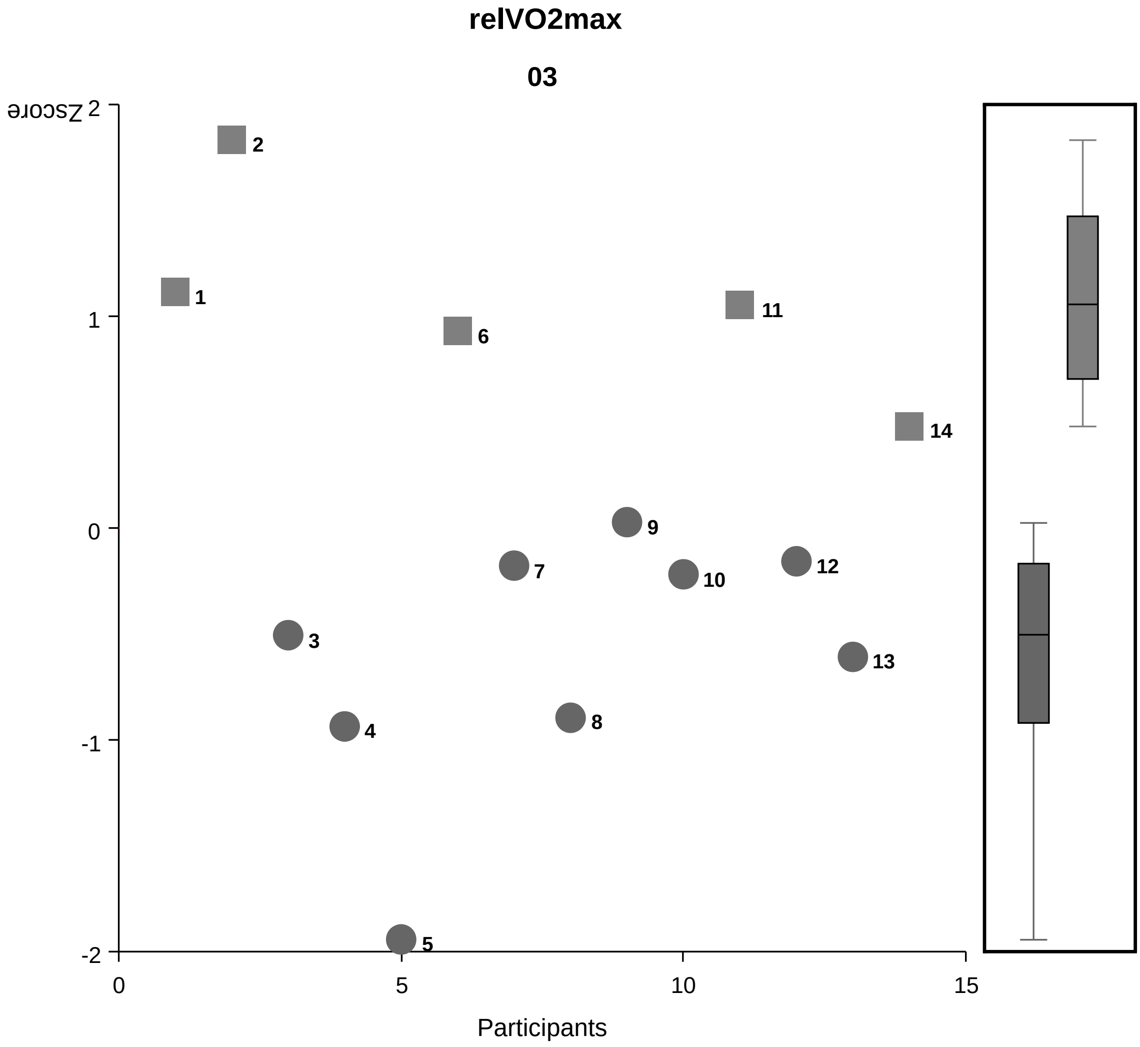

Figure legend: Distribution of Z-score values after 6 months of regular exercise.

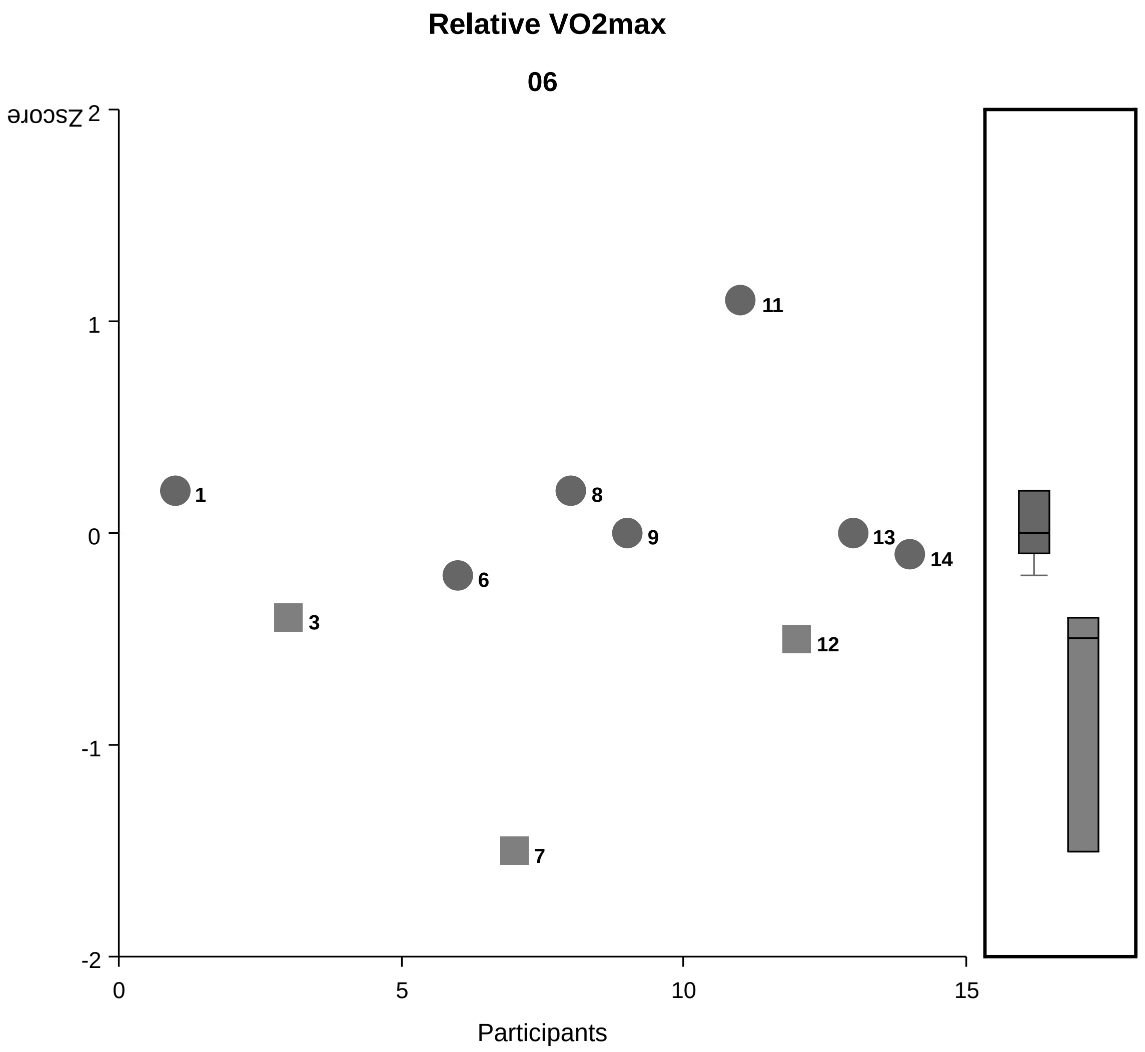

Figure legend: Distribution of Z-score values after 6 months of regular exercise.

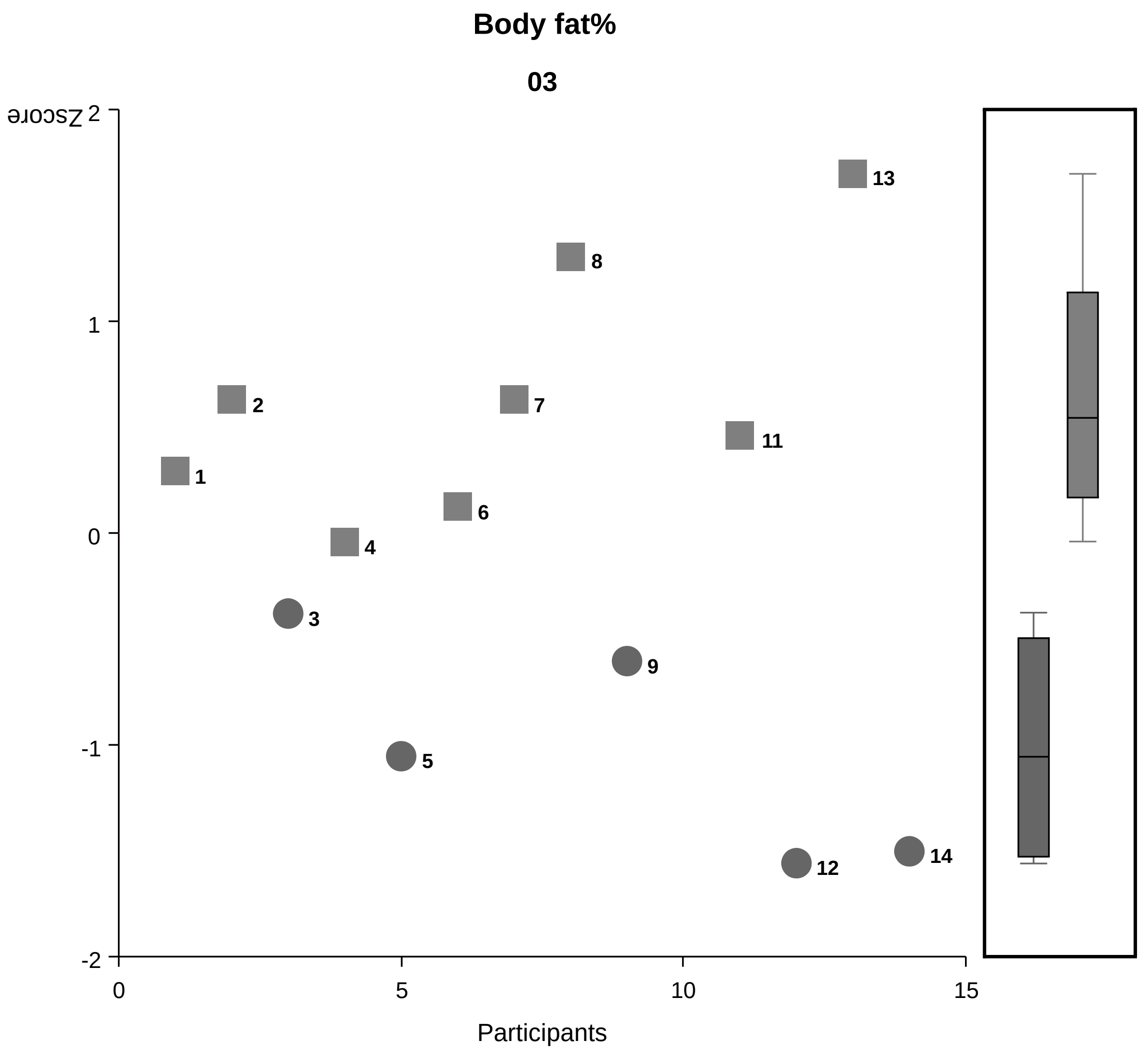

Figure legend: Distribution of Z-score values after 6 months of regular exercise.

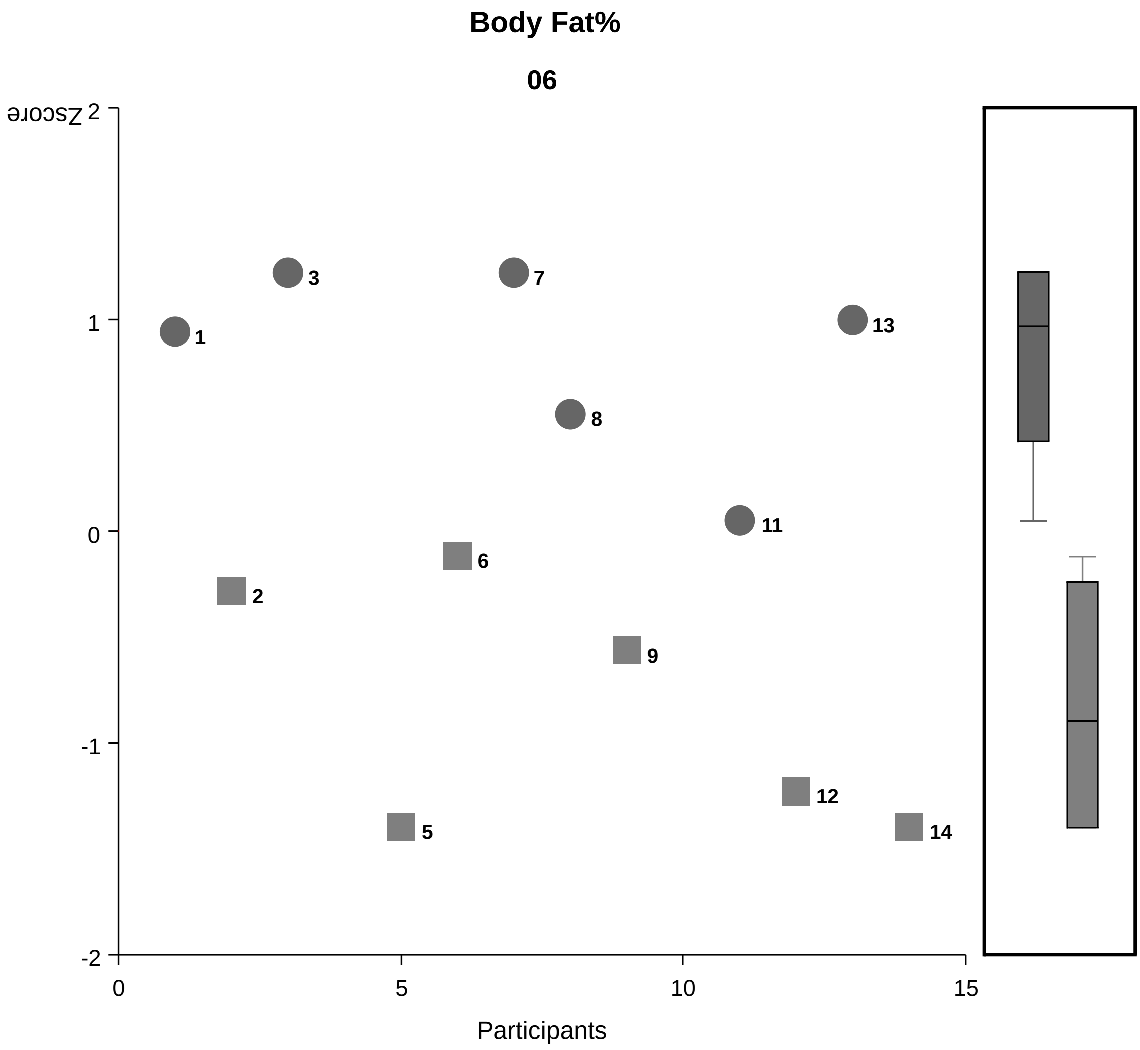

Figure legend: Distribution of Z-score values after 6 months of regular exercise.

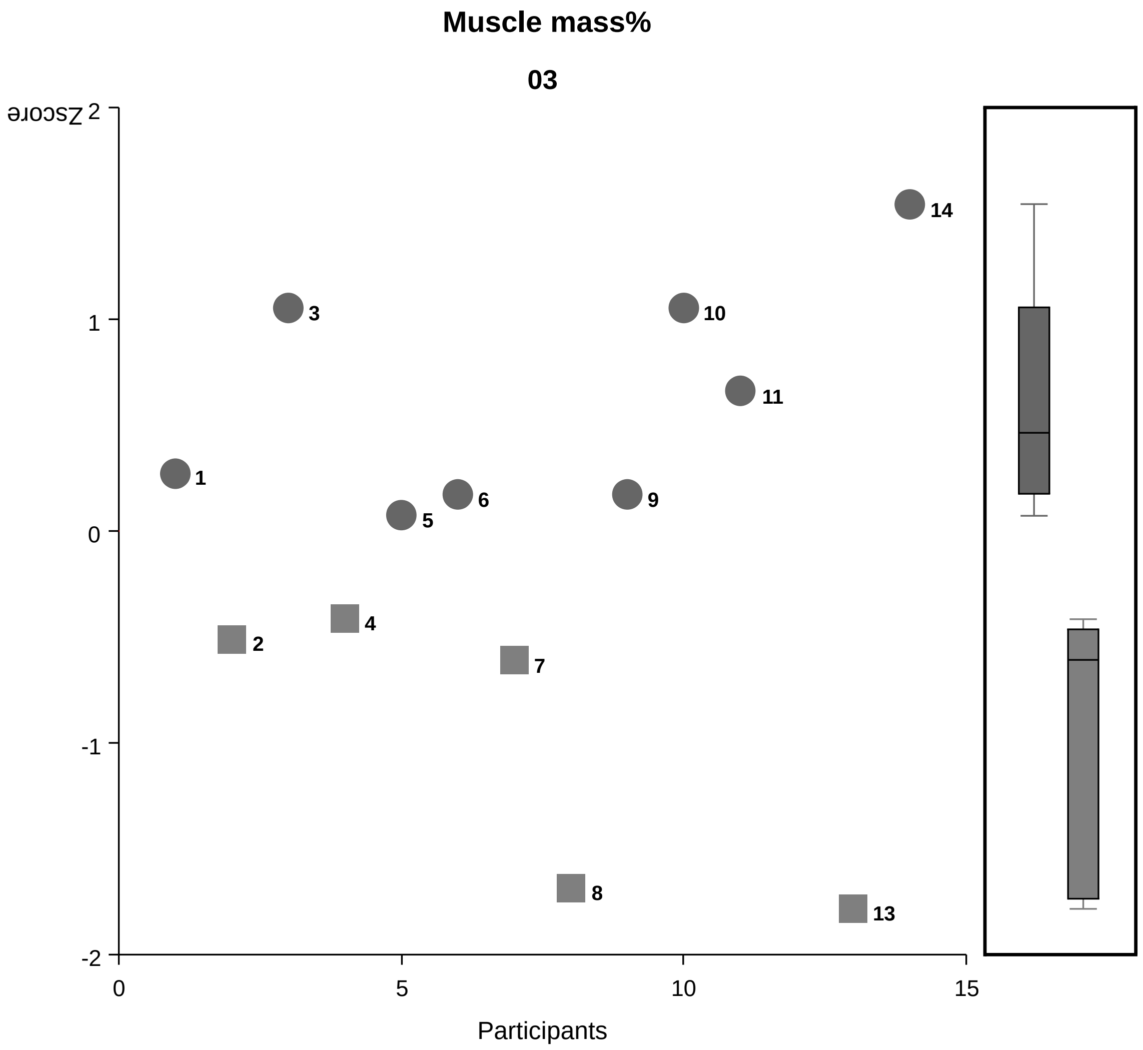

Figure legend: Distribution of Z-score values after 6 months of regular exercise.

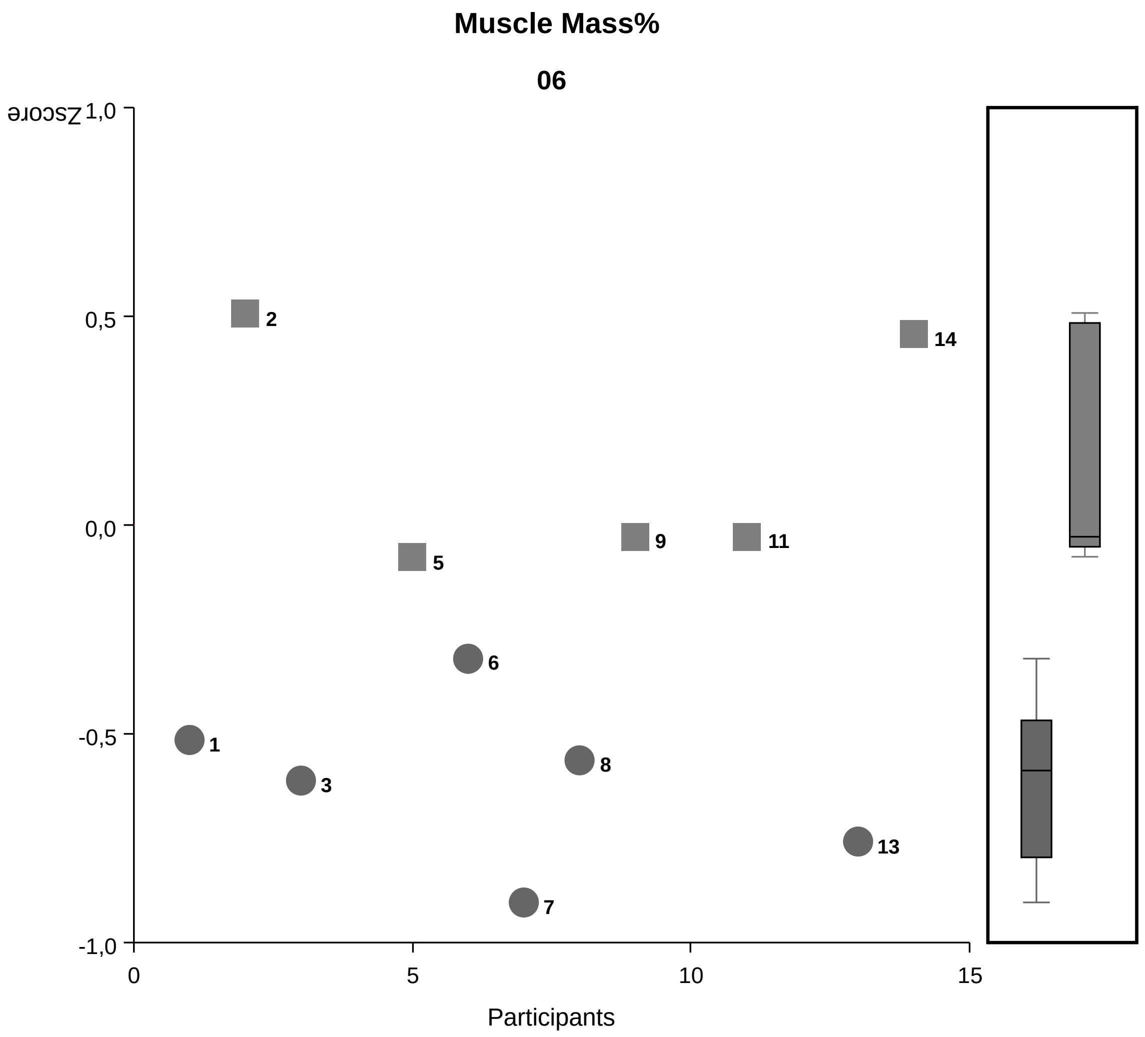

Figure legend: Distribution of Z-score values after 6 months of regular exercise.

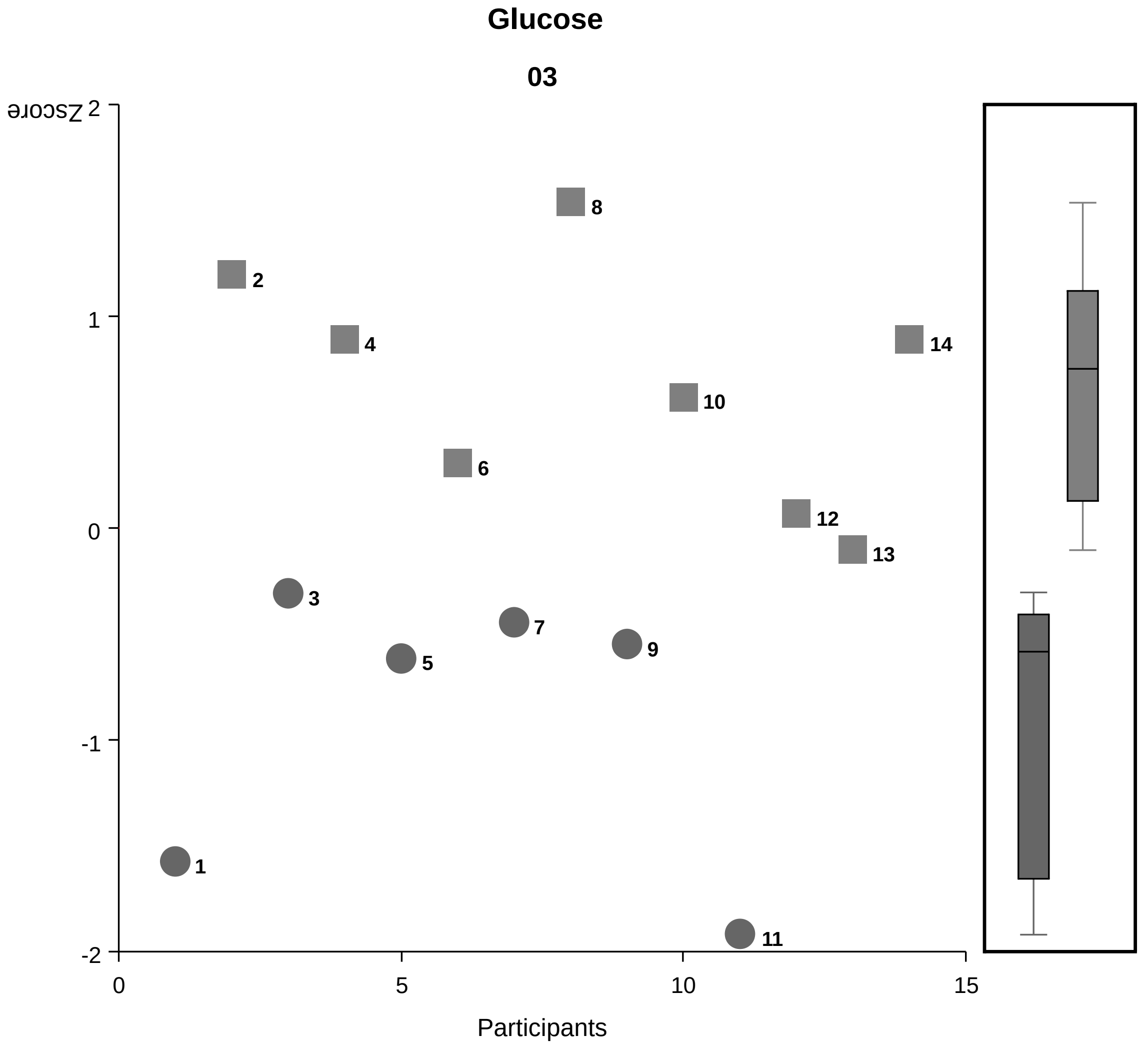

Figure legend: Distribution of Z-score values after 6 months of regular exercise.

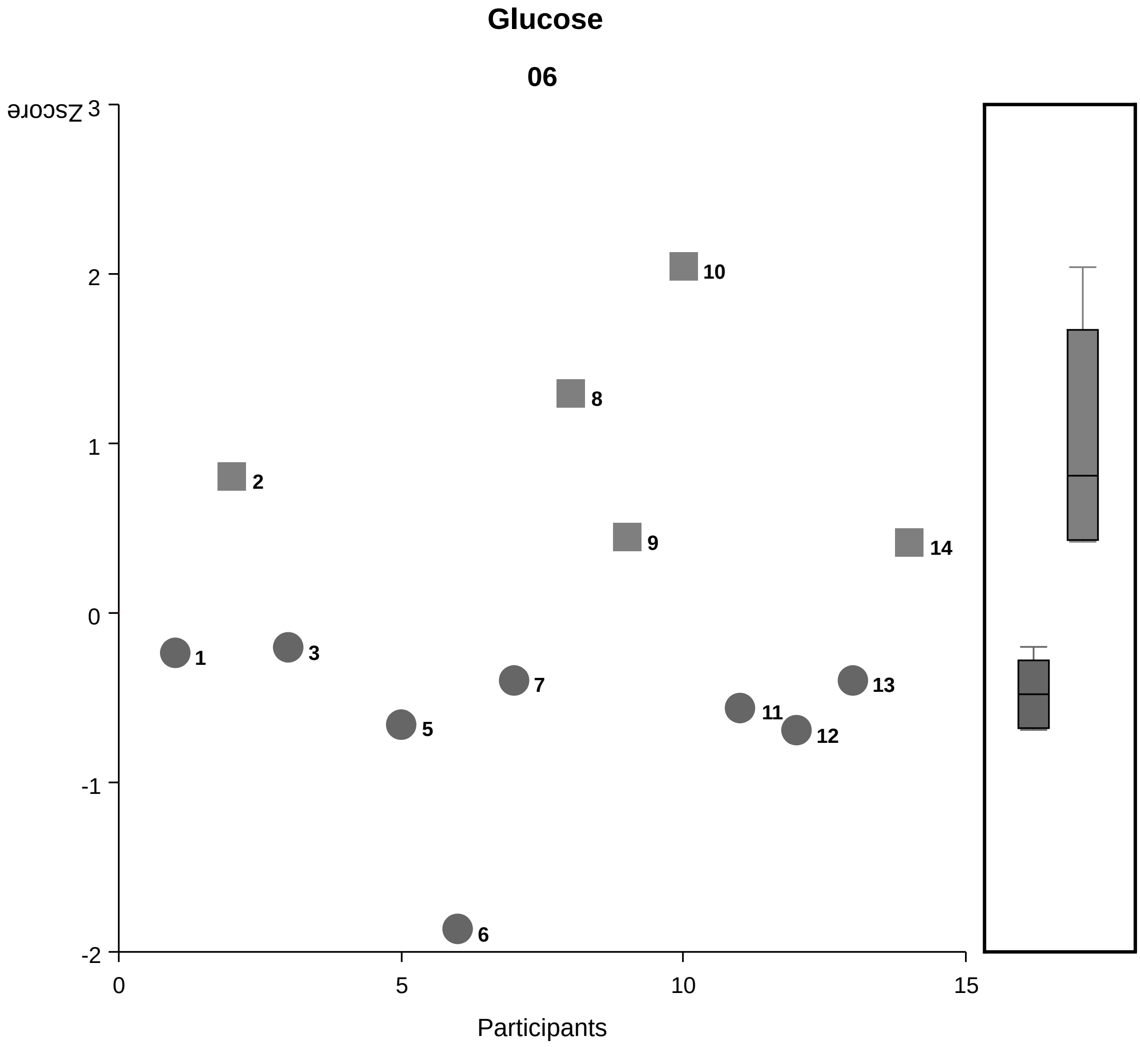

Figure legend: Distribution of Z-score values after 6 months of regular exercise.

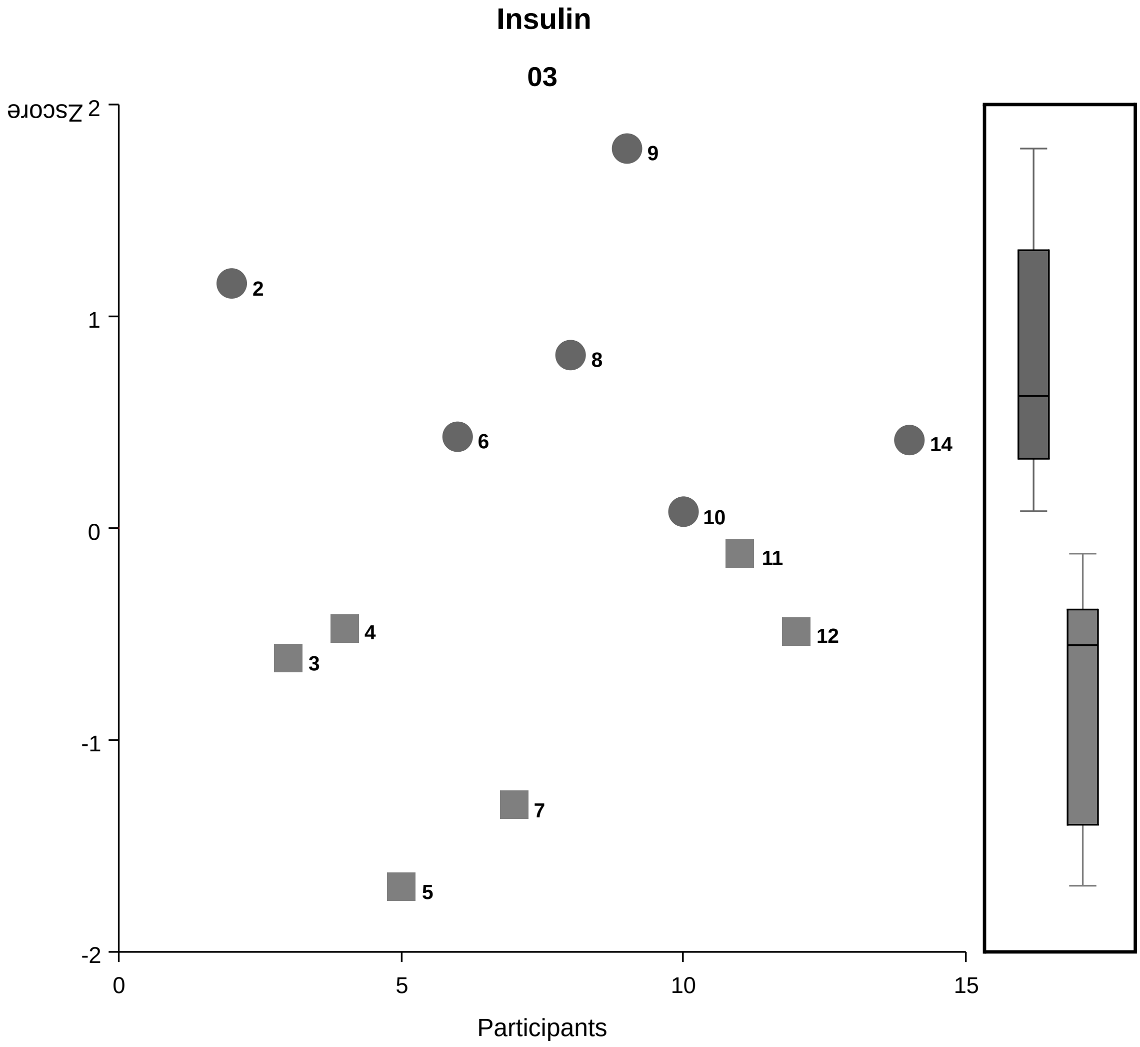

Figure legend: Distribution of Z-score values after 6 months of regular exercise.

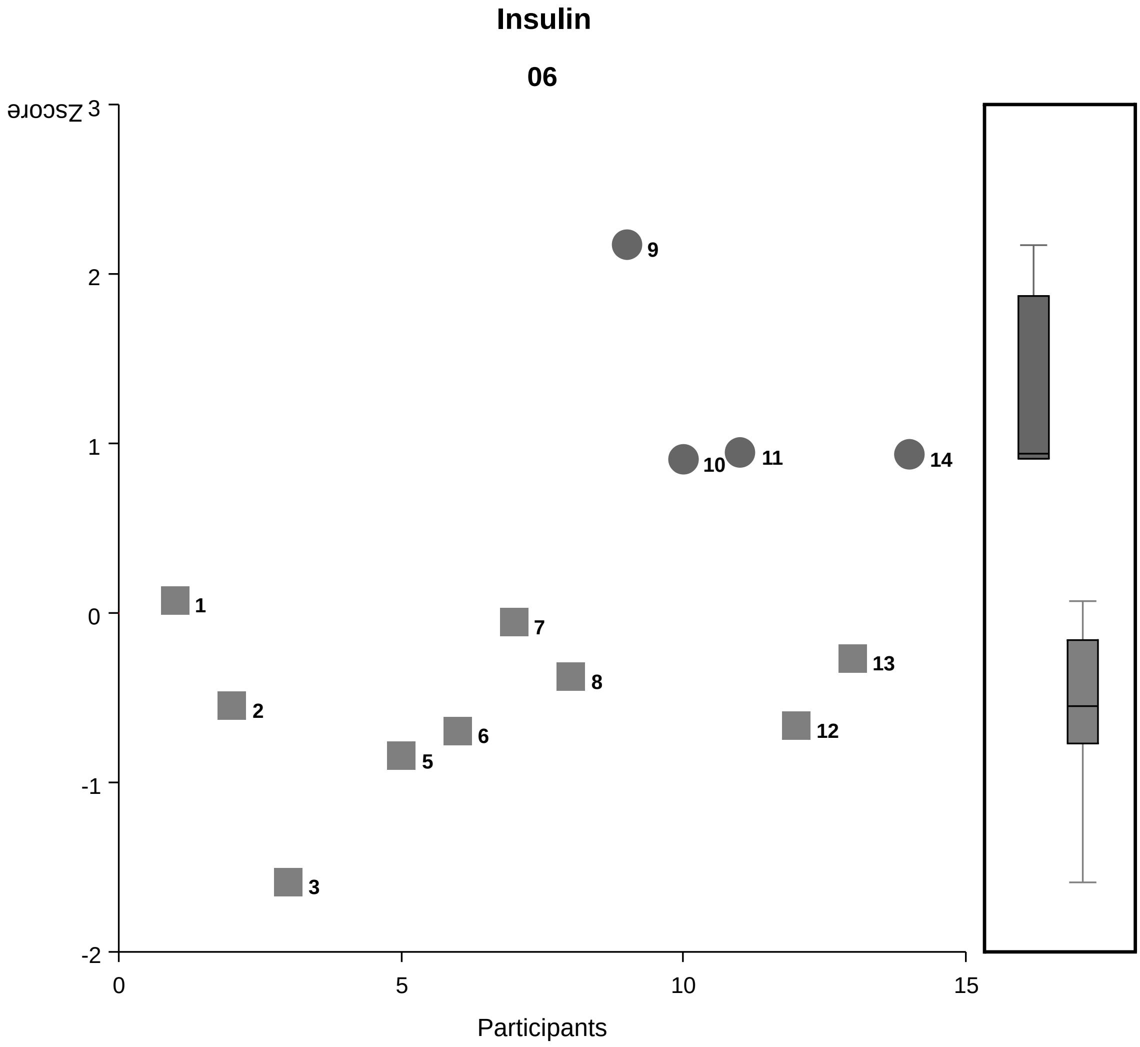

Figure legend: Distribution of Z-score values after 6 months of regular exercise.

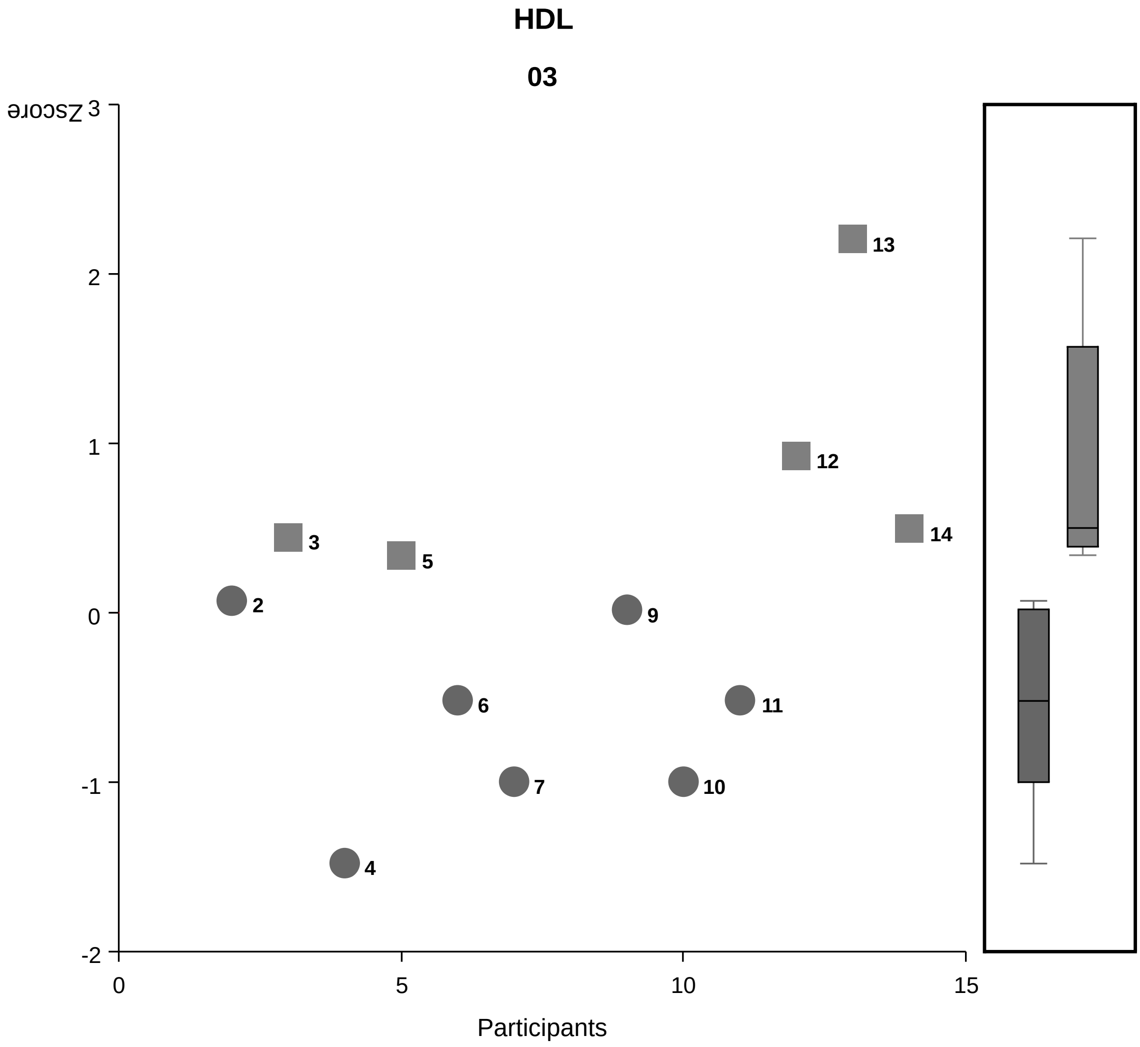

Figure legend: Distribution of Z-score values after 6 months of regular exercise.

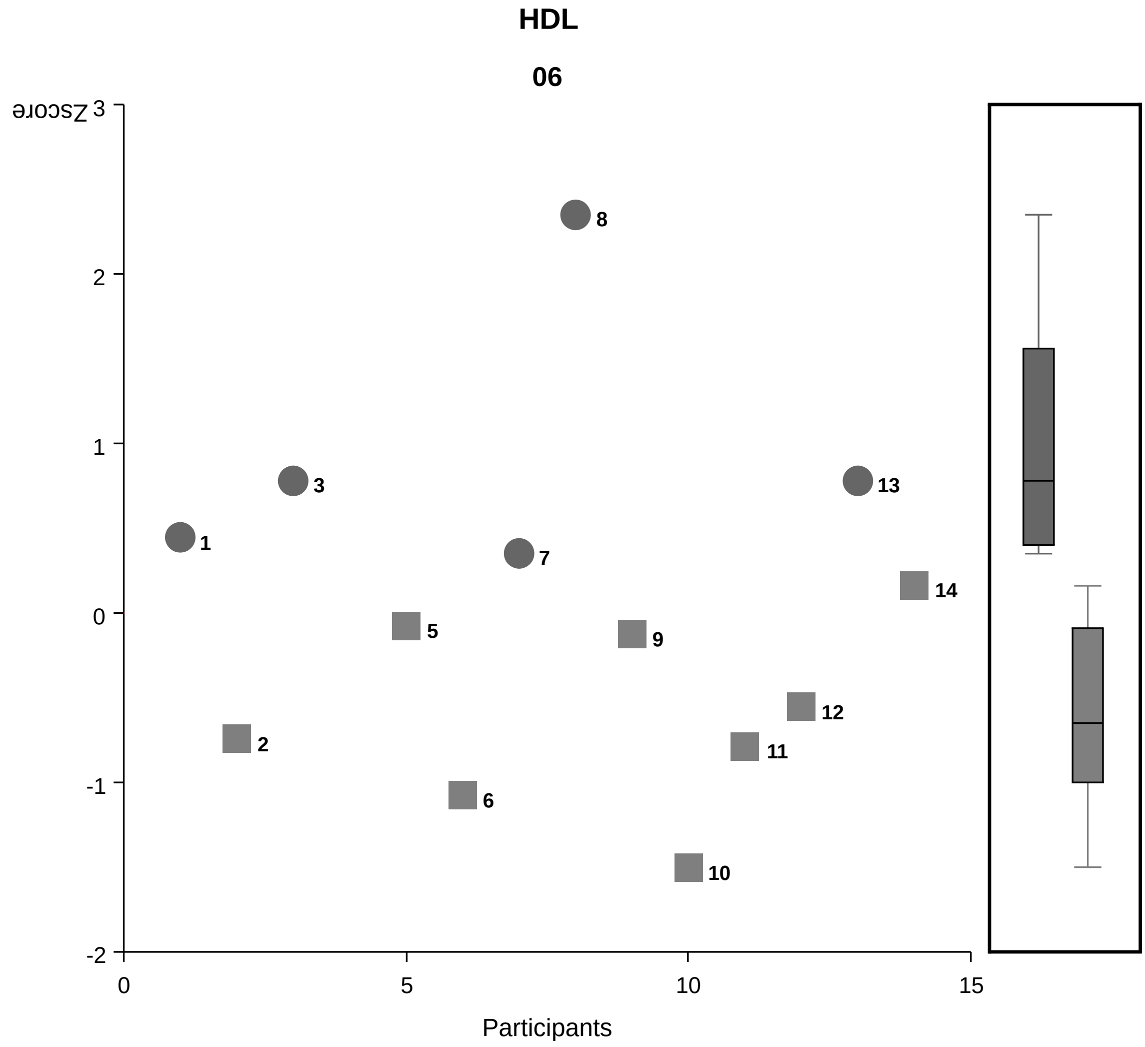

Figure legend: Distribution of Z-score values after 6 months of regular exercise.

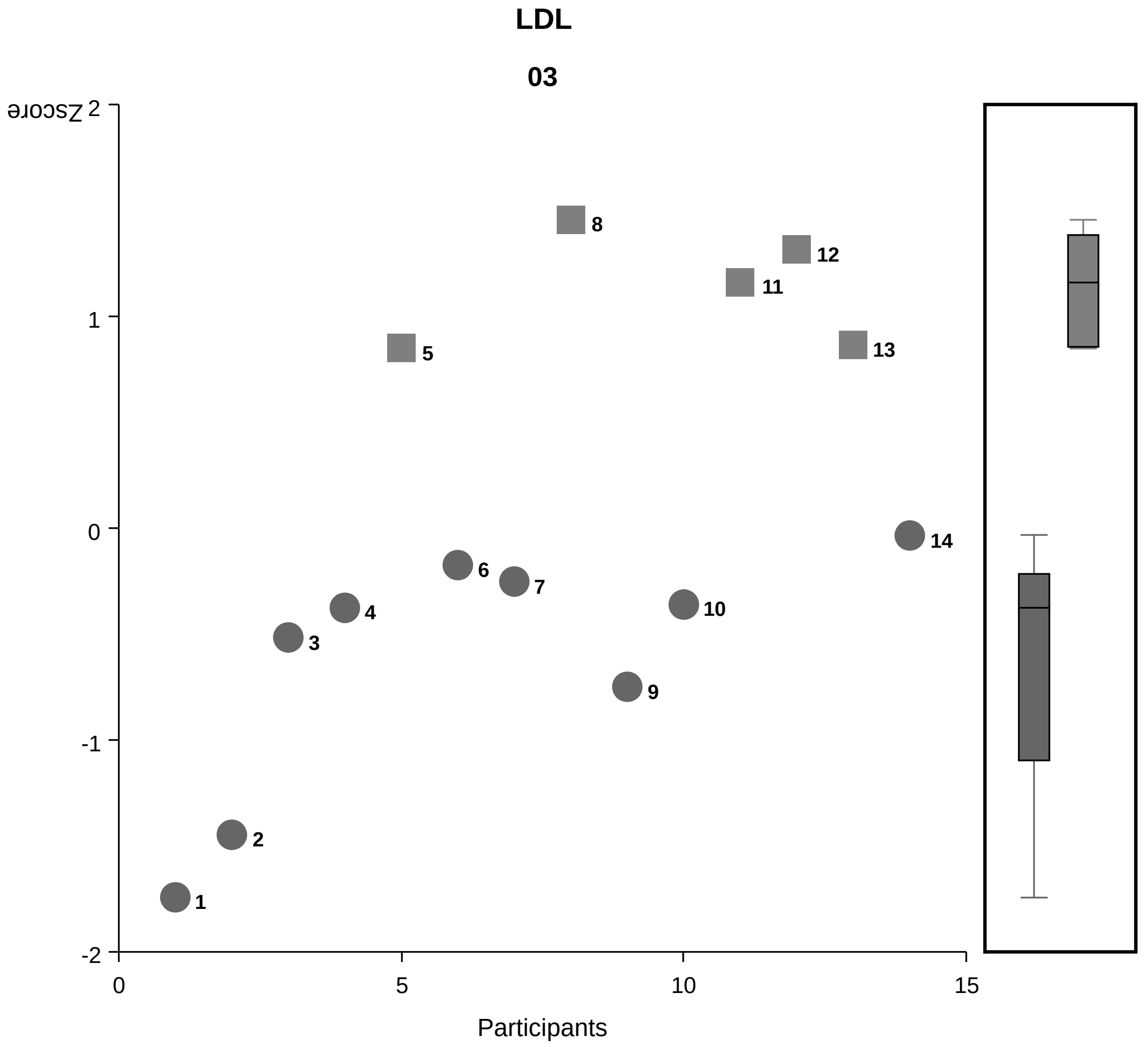

Figure legend: Distribution of Z-score values after 6 months of regular exercise.

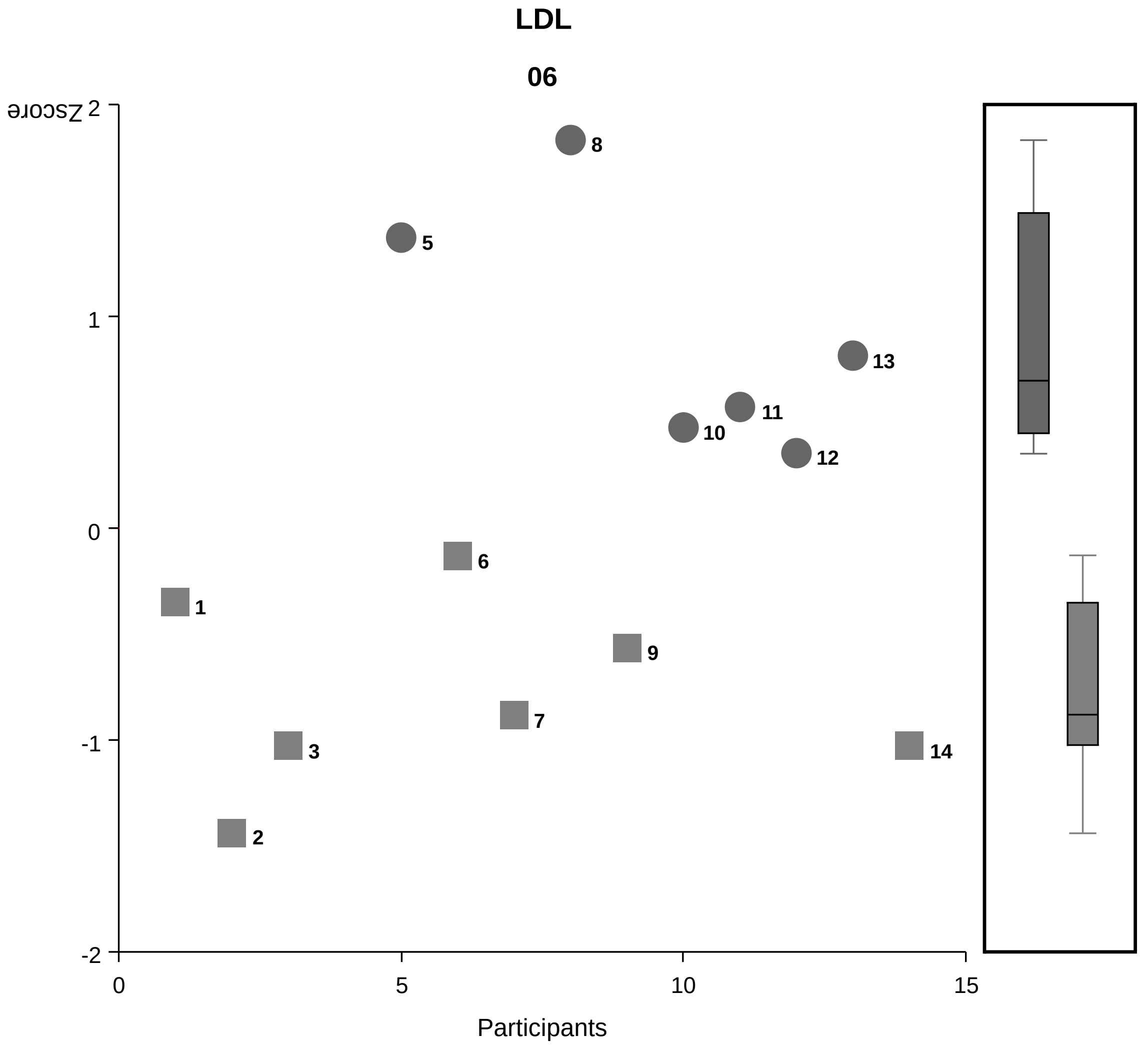

Figure legend: Distribution of Z-score values after 6 months of regular exercise.

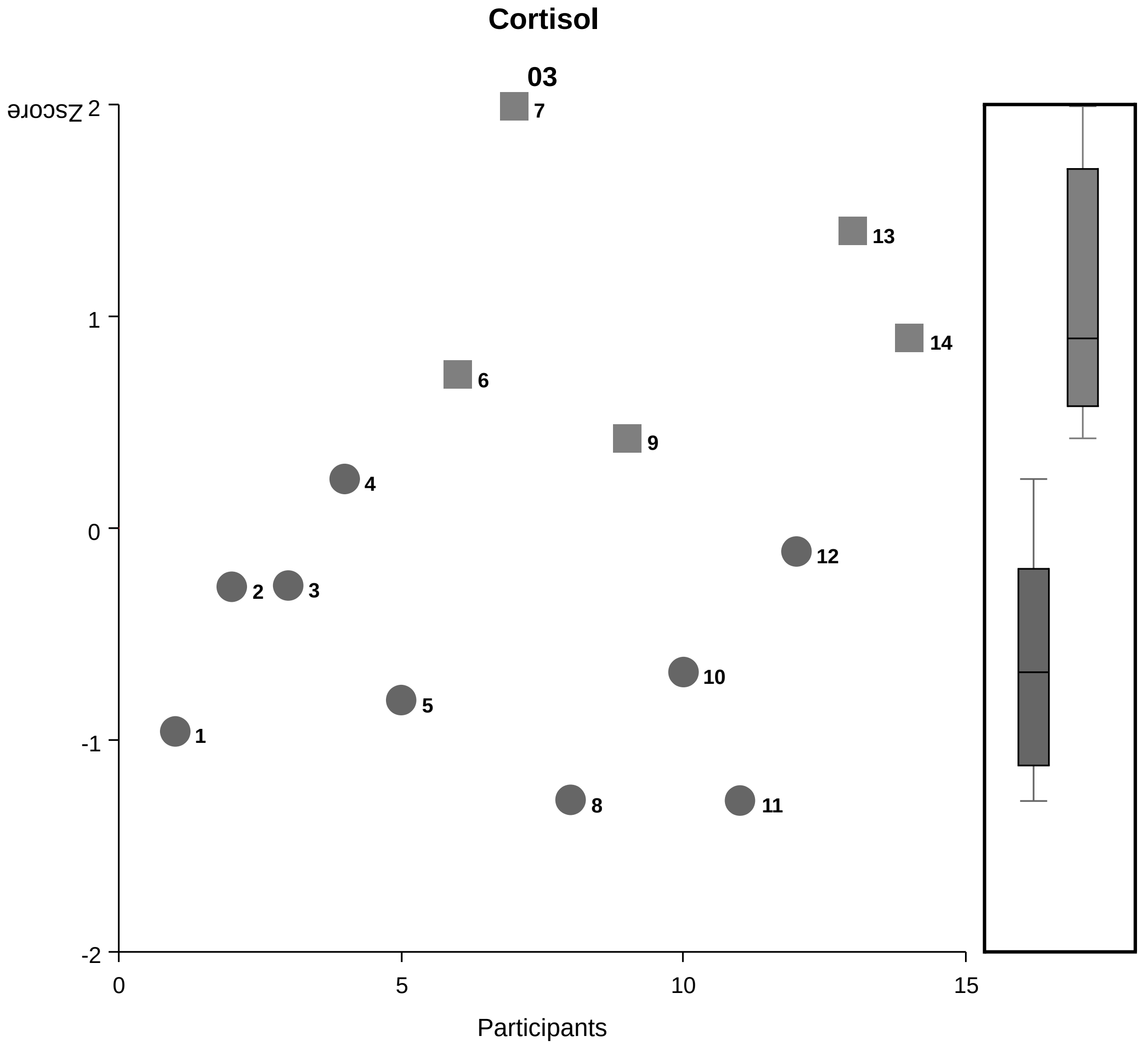

Figure legend: Distribution of Z-score values after 6 months of regular exercise.

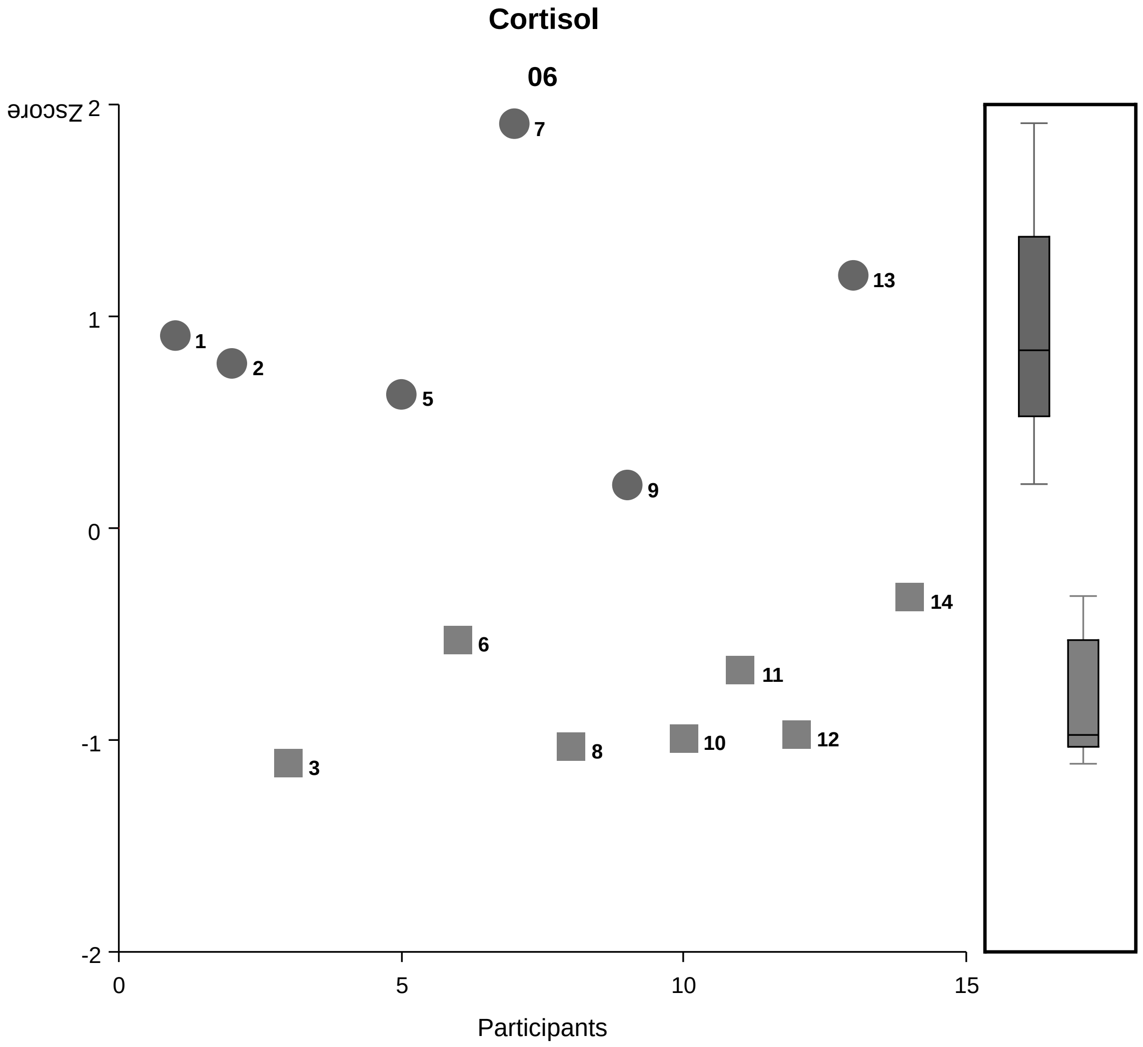

Figure legend: Distribution of Z-score values after 6 months of regular exercise.

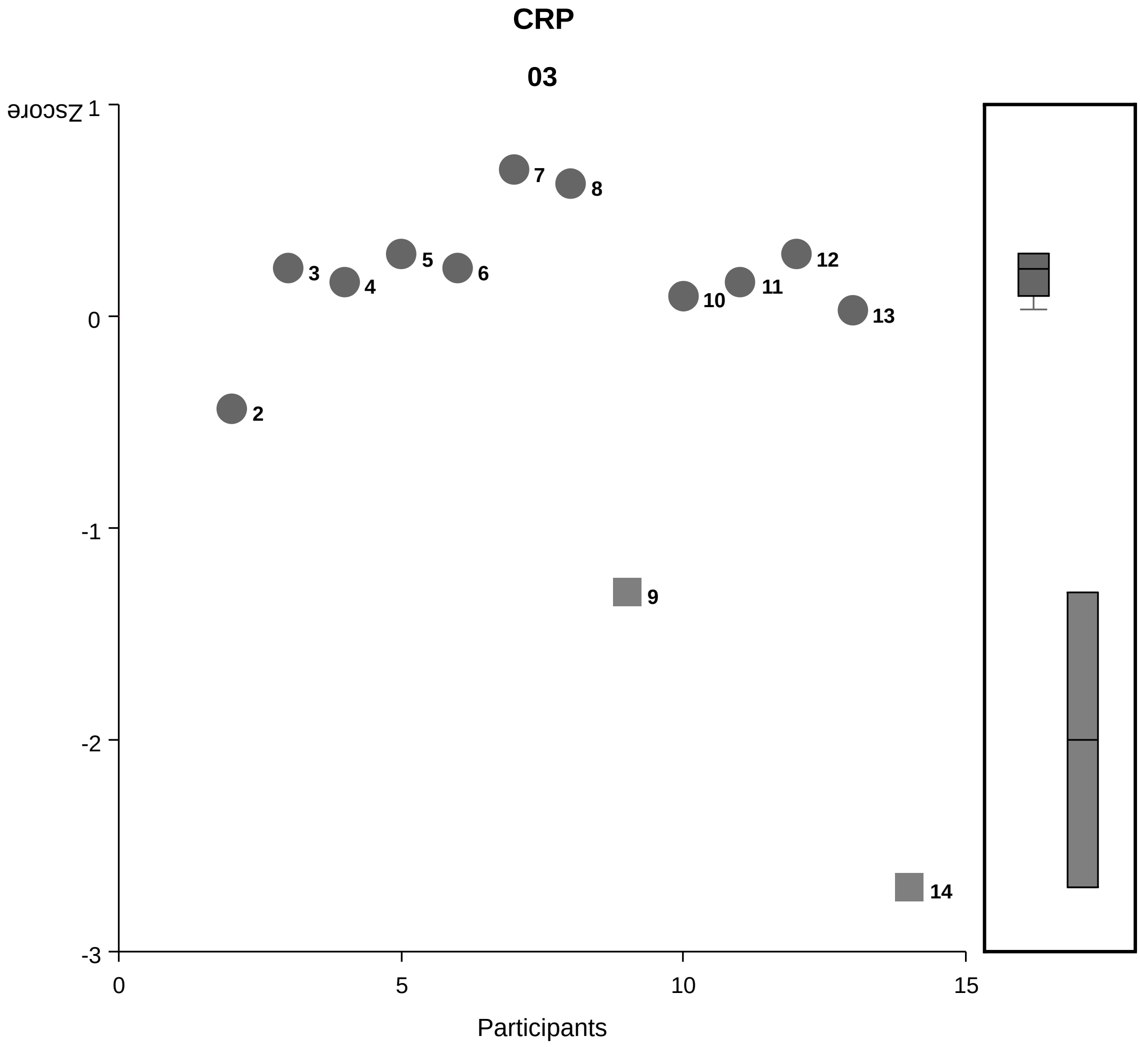

Figure legend: Distribution of Z-score values after 6 months of regular exercise.

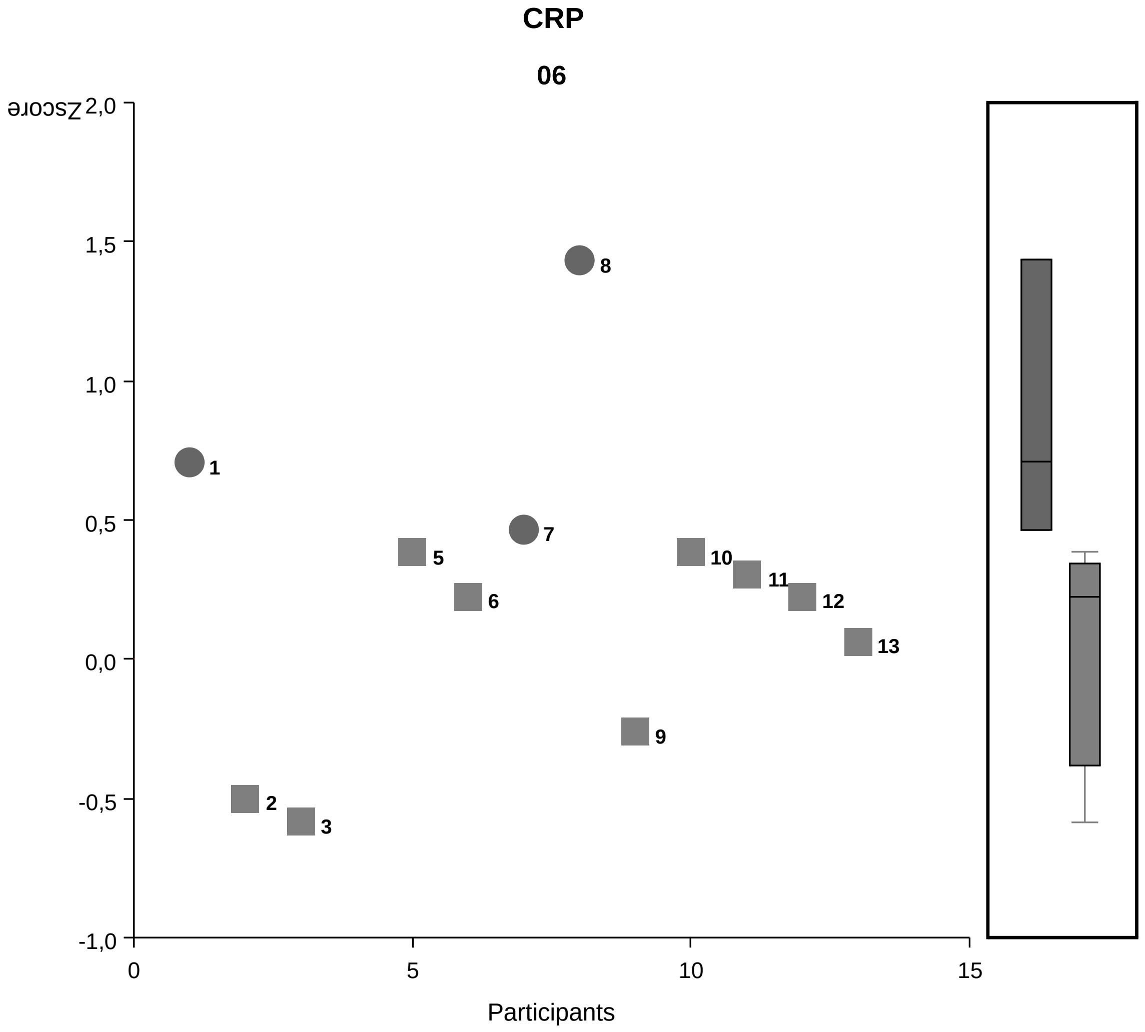

Figure legend: Distribution of Z-score values after 6 months of regular exercise.

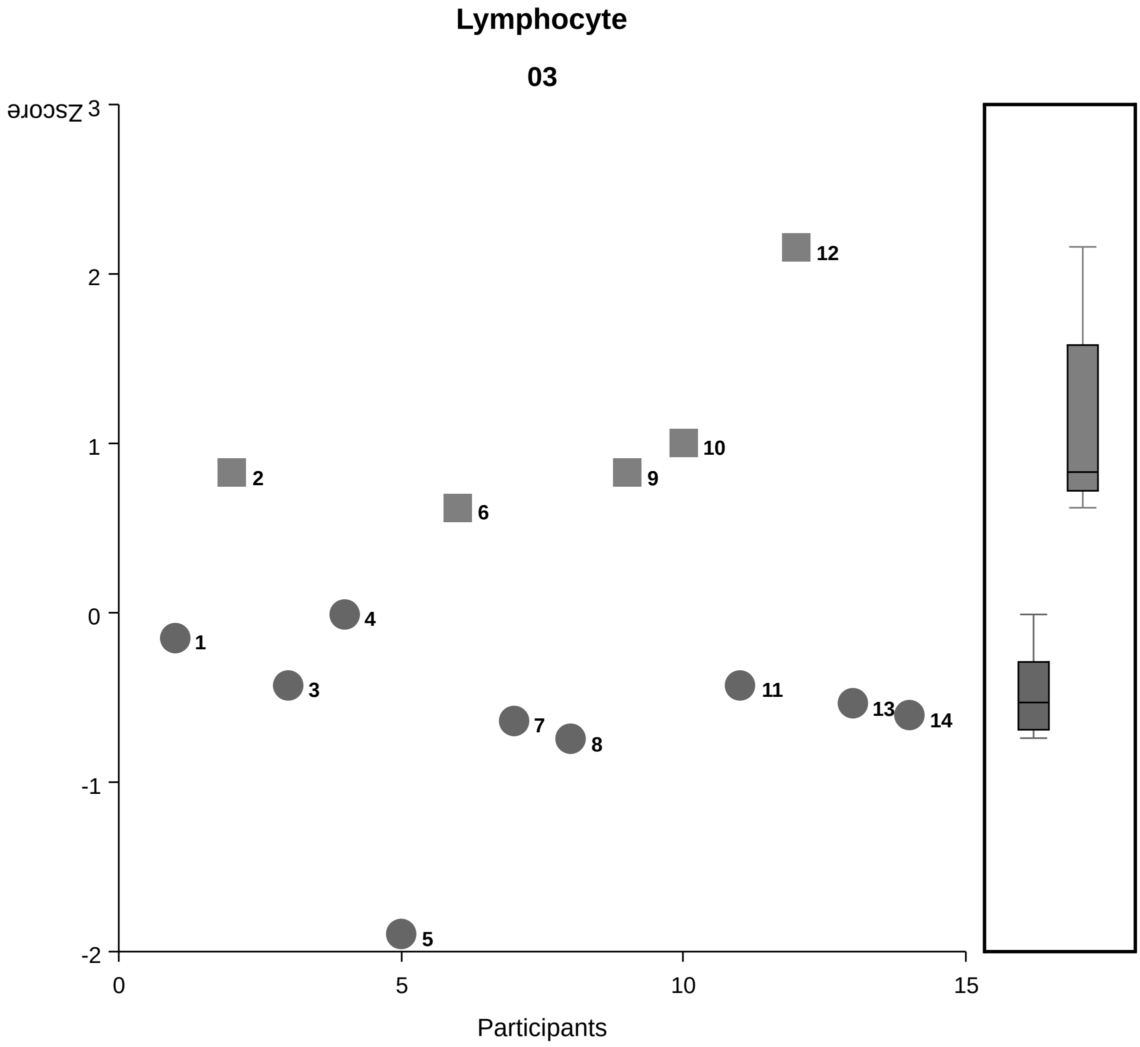

Figure legend: Distribution of Z-score values after 6 months of regular exercise.

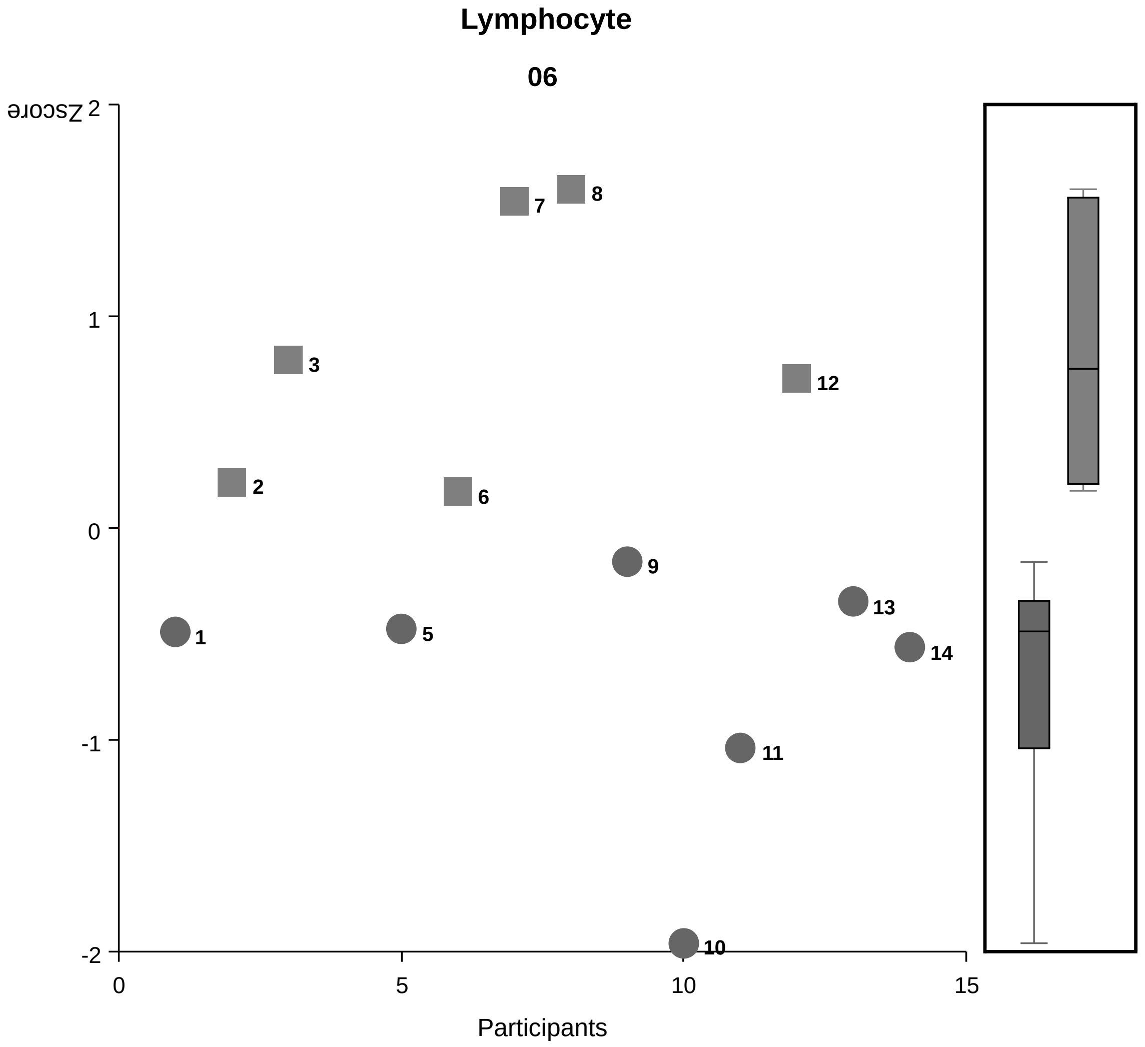

Figure legend: Distribution of Z-score values after 6 months of regular exercise.

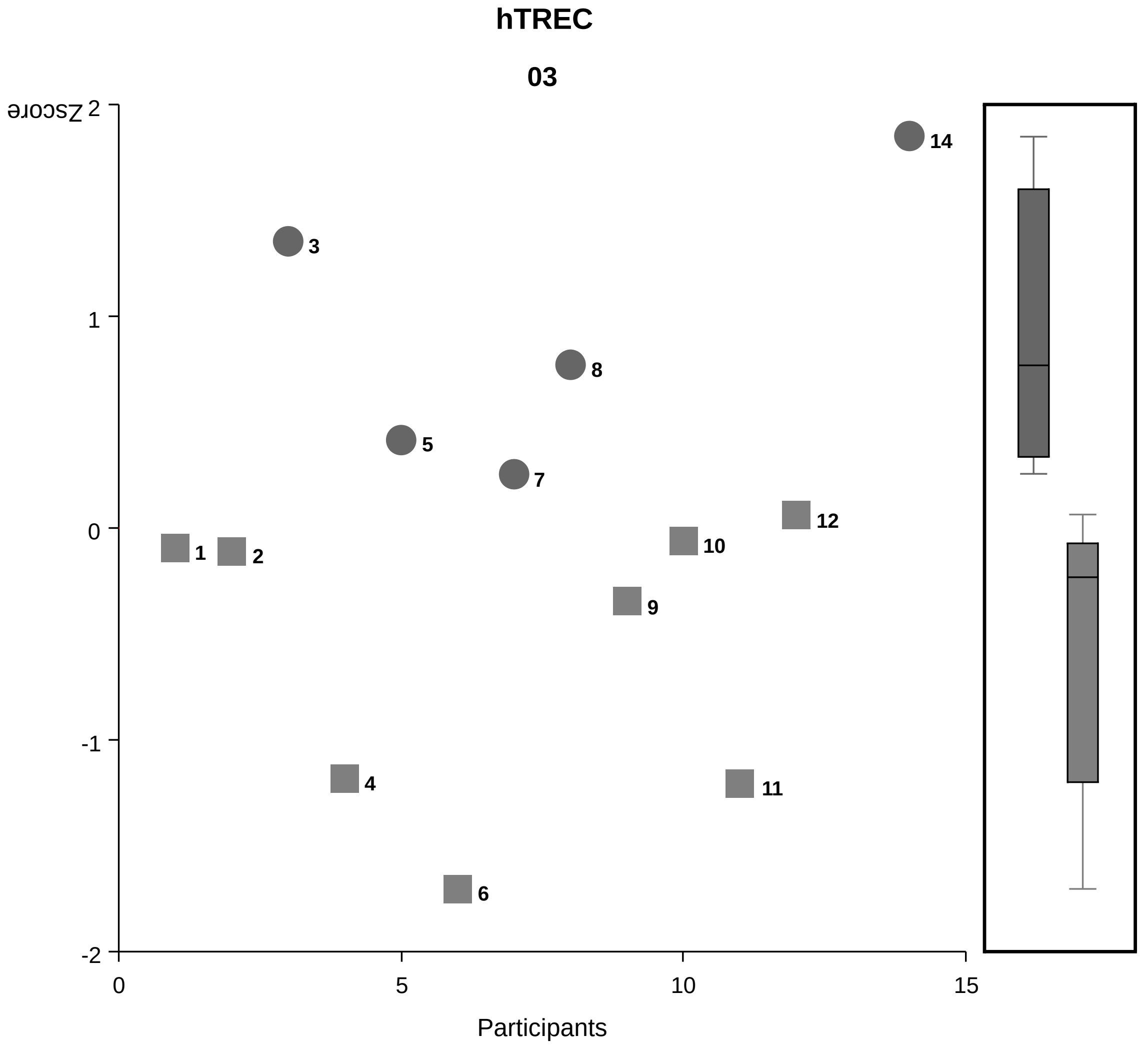

Figure legend: Distribution of Z-score values after 6 months of regular exercise.

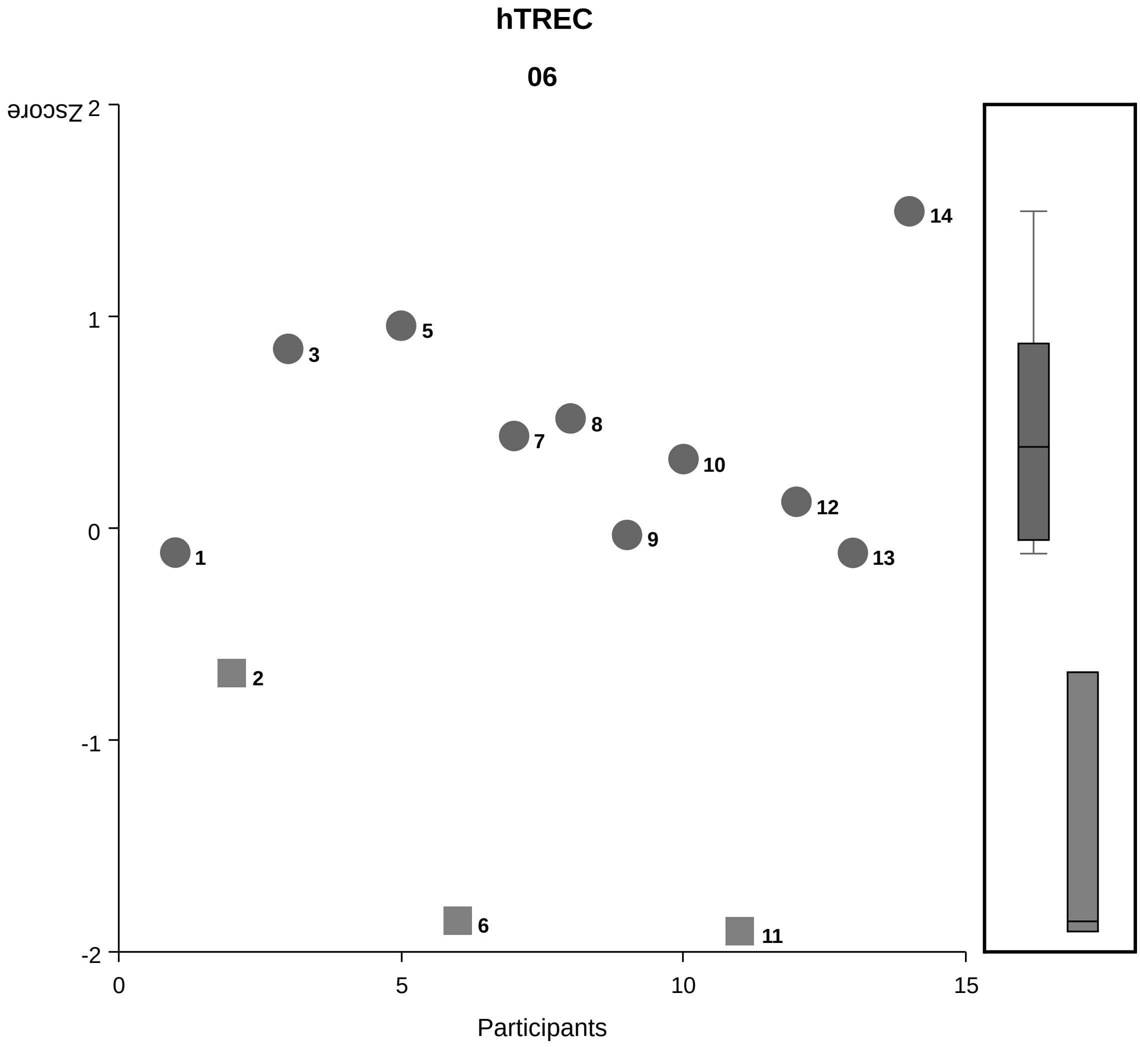

Supplement: Supplementary file 4 [file Data_Sheet_2.docx]
